# Nucleic acid cancer vaccines targeting tumor related angiogenesis. Could mRNA vaccines constitute a game changer?

**DOI:** 10.3389/fimmu.2024.1433185

**Published:** 2024-07-16

**Authors:** Srdan Tadic, Alfredo Martínez

**Affiliations:** Angiogenesis Unit, Oncology Area, Center for Biomedical Research of La Rioja (CIBIR), Logroño, Spain

**Keywords:** cancer, cancer vaccines, anti-angiogenic treatment, mRNA, DNA, tumor vasculature, angiogenesis

## Abstract

Tumor related angiogenesis is an attractive target in cancer therapeutic research due to its crucial role in tumor growth, invasion, and metastasis. Different agents were developed aiming to inhibit this process; however they had limited success. Cancer vaccines could be a promising tool in anti-cancer/anti-angiogenic therapy. Cancer vaccines aim to initiate an immune response against cancer cells upon presentation of tumor antigens which hopefully will result in the eradication of disease and prevention of its recurrence by inducing an efficient and long-lasting immune response. Different vaccine constructs have been developed to achieve this and they could include either protein-based or nucleic acid-based vaccines. Nucleic acid vaccines are simple and relatively easy to produce, with high efficiency and safety, thus prompting a high interest in the field. Different DNA vaccines have been developed to target crucial regulators of tumor angiogenesis. Most of them were successful in pre-clinical studies, mostly when used in combination with other therapeutics, but had limited success in the clinic. Apparently, different tumor evasion mechanisms and reduced immunogenicity still limit the potential of these vaccines and there is plenty of room for improvement. Nowadays, mRNA cancer vaccines are making remarkable progress due to improvements in the manufacturing technology and represent a powerful potential alternative. Apart from their efficiency, mRNA vaccines are simple and cheap to produce, can encompass multiple targets simultaneously, and can be quickly transferred from bench to bedside. mRNA vaccines have already accomplished amazing results in cancer clinical trials, thus ensuring a bright future in the field, although no anti-angiogenic mRNA vaccines have been described yet. This review aims to describe recent advances in anti-angiogenic DNA vaccine therapy and to provide perspectives for use of revolutionary approaches such are mRNA vaccines for anti-angiogenic treatments.

## 1 Introduction

Cancer is the leading cause of death in the world, with about 10 million people estimated to have died from this disease in 2022. The most lethal cancer types were the ones affecting the lung, colon, liver, stomach, and breast (1). Estimates, according to the American Cancer Society, indicate that by 2040 the number of cases will reach approximately 29 million if the current progression rate remains (2). Cancer is defined as a group of diseases that can affect any part of the body and is characterized by a group of cells that escape from the body´s control and proliferate constantly while being refractory to the regulatory signals. Unique features of this disease, the so-called hallmarks of cancer, explain cancer cells´ ability to propagate, survive, grow, invade, and metastasize (3).

Metastasis is one of the most prominent and complex cancer hallmarks. Metastasis is the ability of some cancer cells to invade and colonize a distant tissue or organ. Metastatic disease is responsible for more than 90% of deaths in cancer patients (4). Therefore, metastatic disease is the number one cancer-related health emergency issue worldwide that needs an adequate solution. One of the crucial processes required for metastasis initiation is angiogenesis (5). Angiogenesis implies the formation of new blood vessels from established vascular beds with the help of endothelial and stromal cells, and represents another critical cancer hallmark (6).

The establishment of new vasculature supports tumor growth, invasion, and metastasis in different ways. The process of angiogenesis is regulated by a complex interaction between pro-angiogenic and anti-angiogenic factors. Vascular endothelial growth factor (VEGF), platelet derived growth factor (PDGF), hepatocyte growth factor (HGF), basic fibroblast growth factor (bFGF), tumor growth factor β (TGF-β), or adrenomedullin (AM) are some characteristic pro-angiogenic growth factors. Beside them, matrix metalloproteinases (MMP) and angiopoietins are other important molecules with pro-angiogenic functions. On the other hand, some of the important anti-angiogenic factors include trombospondin 1, endo- and angio- statin (7, 8).

In normal physiological conditions, a balance between pro- and anti- angiogenic factors is reached, thus the vasculature of most adult tissues is relatively quiescent and stable (9). However, in pathological conditions, such as cancer, the situation is drastically different. Tumor development is a rapid and chaotic process in which tumor cells grow abnormally and invade surrounding tissue resulting in its destruction (10). As a consequence, cells are exposed to high metabolic stress in terms of hypoxia, shortage of glucose, but also mechanical stress due to the pressure applied on the tissue by expanding tumor cells (11, 12).

Hypoxia (low oxygen levels) occurs due to increased demand and/or decreased supply of oxygen to a specific tissue and represents the major mechanisms behind angiogenesis induction (13). The levels of oxygen are tissue specific and therefore hypoxia can be relative throughout the body, however when the standard oxygen levels for the specific tissue markedly drop, hypoxia is established (14). The main cellular sensors of hypoxia are the hypoxia-inducible factors (HIFs) and, more specifically, the protein HIF-1α. Under normoxic conditions, HIF-1α is rapidly degraded. On the other hand, under hypoxic conditions, HIF-1α accumulates in the cytoplasm, transfers into the nucleus where it binds HIF-1β forming an heterodimeric transcription factor (15). This transcription factor binds to specific sites (called hypoxia response elements or HREs) in the promotor regions of particular genes involved in angiogenesis promotion, such as *VEGF*, angiopoietins 1 and 2, *PDGF*, *bFGF* or *AM*, up-regulating their expression. All these factors activate endothelial cells through specific membrane receptors. This activation leads to endothelial cell proliferation, migration, sprouting, adhesion and finally resulting in the formation of new blood vessels (16, 17).

**Figure 1:**
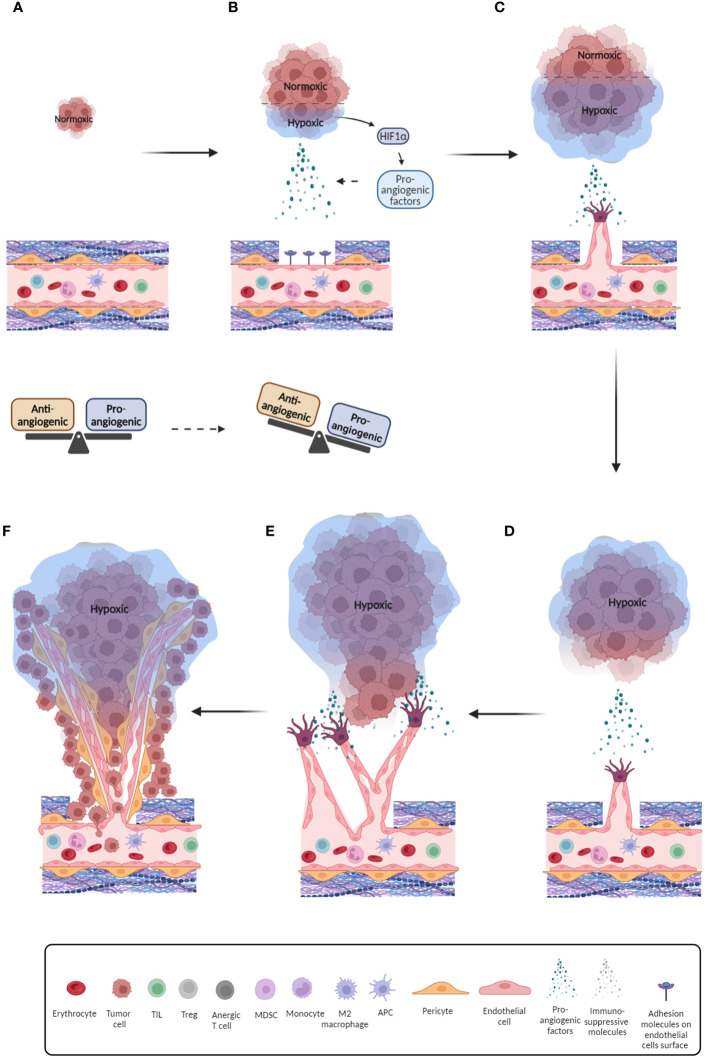
Schematic overview of the tumor-related angiogenesis process. **(A)** Initial phase of tumor formation with endothelium in balanced state; **(B)** Further development of tumor and onset of hypoxia. As a consequence, pro-angiogenic factors are released and balance of pro- and anti-angiogenic factors is breached; **(C)** Pro-angiogenic factors bind to their receptors on endothelial cells and initiate their recruitment and migration by inducing their differentiation and release of proteases that digest the extracellular matrix, thus allowing endothelial cells to detach and migrate; **(D)** Further development of tumor, promotion of hypoxic conditions and increased secretion of pro-angiogenic factors. Endothelial tip cells move towards pro-angiogenic signals; **(E)** Formation of initial blood vessels around the tumor; **(F)** Formation of aberrant blood vessels with irregular blood flow and reduced pericyte coverage which further promotes hypoxia. Furthermore, this provides space for release of tumor cells into blood vessels and metastasis initiation.

Tumor growth and metastasis are high energy demanding processes, so they require increased nutrient and oxygen availability and therefore high vascularity (18). Nevertheless, although the process of angiogenesis initiated in tumors aims to support tumor survival and progression, the over-signaling by pro-angiogenic factors often promotes rapid and uncontrollable formation of blood vessels. Therefore, tumor blood networks often fail to properly mature, divide and form adequate size vessels, which results in malformations of blood vessels and chaotic blood flow (19). This uneven blood flow establishes chronic, acute, or cyclic hypoxia that acts as a positive feedback loop that up-regulates pro-angiogenic factors, further promoting angiogenesis. Additionally, these vessels can be highly permeable and feature increased fluid pressure as a consequence of endothelial junction disruption (20). These features provide perfect conditions to allow tumor cells to disseminate, invade surrounding tissue, and metastasize (**Figure 1**).

**Figure 2:**
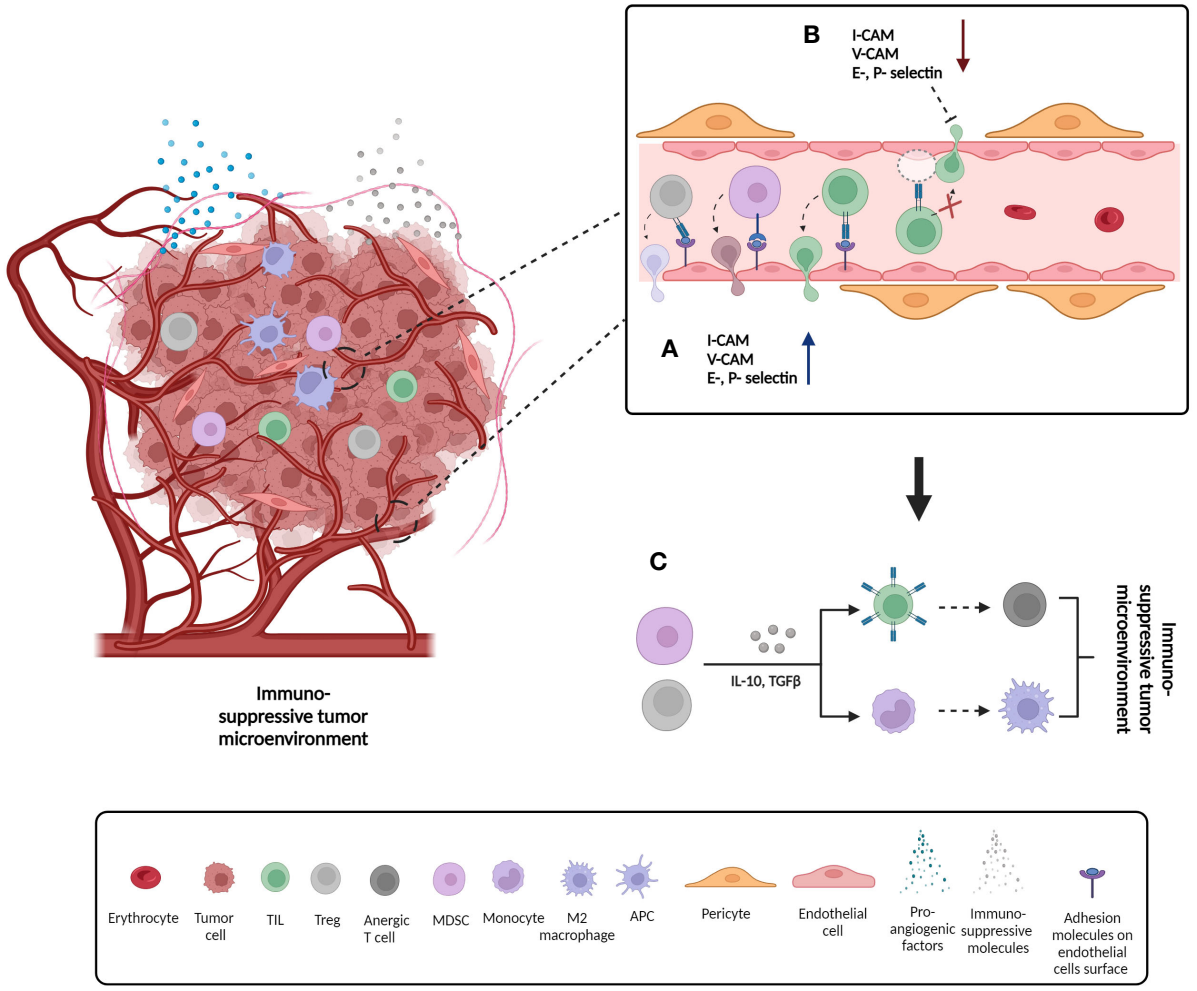
Formation of immunosuppressive tumor microenvironment as a consequence of changes on endothelial cells´ surface adhesion molecules expression. **(A)** Up-regulation of adhesion molecule expression on the surface of endothelial cells and consequent infiltration of pro-inflammatory cells such as tumor infiltrating lymphocytes (TILs), but also anti-inflammatory cells such as regulator T cells (Tregs) and myeloid derived T cells (MDSCs); **(B)** Down-regulation of adhesion molecule expression on the surface of endothelial cells and consequent prevention of cell infiltration; **(C)** Some of the mechanisms by which tumor suppressor cells such as Treg and MDSCs promote formation of immunosuppressive tumor microenvironment. Secretion of immunosuppressive signals such as IL-10 and TGFβ can cause anergy of infiltrating T cells and therefore loss of function. In addition, the same signals can induce differentiation of monocytes to immunosuppressive M2 macrophage phenotype. IL10: Interleukin 10, TGFβ: tumor growth factor beta.

Besides promoting morphological changes on tumor blood vessels that lead to tumor growth and metastasis, pro-angiogenic factors can alter the state of the vascular tissue by influencing the endothelial cell´s immune profile. Endothelial cells regulate the expression of adhesion molecules on blood vessels in accordance with inflammatory processes in the tissue (21). For example, a constant pro-angiogenic signaling can induce so-called endothelial cell anergy and down-regulation of endothelial cell adhesion molecules, such as intracellular adhesion molecule 1 and 2 (ICAM-1 and-2), vascular cell adhesion molecule 1 (VCAM-1), E- and P- selectin. These molecules are necessary for the establishment of the interactions between leukocytes and the vessel´s wall that promote extravasation and infiltration of leukocytes, especially tumor-infiltrating lymphocytes (TILs). TILs are crucial mediators of anti-tumor immune response and their presence correlates with a positive outcome in patients suffering from a variety of tumor types (22). Downregulation of endothelial adhesion molecules directly prevents TILs from reaching the tumor and thus constitutes a mechanism of anti-tumor immune response abrogation (23, 24). On the other hand, the pro-angiogenic stimulation can up-regulate the expression of some adhesion molecules thus allowing infiltration of pro-inflammatory cells, but also infiltration of regulatory T cells (Treg) and myeloid derived suppressor cells (MDSCs) thus contributing to chronic inflammation and an immune suppressive environment, both of which are associated with bad prognosis (25, 26). The above-mentioned features emphasize the important role of angiogenesis in cancer growth and metastasis, thus pointing this process as an attractive target for anti-tumor therapies (**Figure 2**).

## 2 Anti-angiogenic pharmacological therapies

Different drugs have been developed to target angiogenesis and some of them are currently recognized as standard-of-care, usually in combination with targeted therapies, and/or chemo- and radio- therapies (27). Back in the 1970s, Judah Folkman was the pioneer in tumor angiogenesis research and proposed that tumors cannot grow behind certain limit if the blood supply is blocked. Therefore he proposed a targeted therapy that would result in disruption of the tumor blood vessels, prevention of their new formation, and thus induction of tumor cell death (28). This led to the development of targeted therapies that included monoclonal antibodies, tyrosine kinase inhibitors (TKI), receptor fusion proteins, and several others.

These therapies were developed to target pro-angiogenic factors and/or their receptors and to induce their down-regulation (29). This approach provided promising results in targeting tumor related angiogenesis and led to the clinical approval of some of these agents, including bevacizumab, a monoclonal antibody targeting VEGFA which is approved for use in the treatment of multiple solid tumor types, such as colorectal, lung, renal, cervical, ovarian cancer, and glioblastoma, either alone or in combination with other drugs (30). Sorafenib and sunitinib are TKIs that were approved for use in the clinical setting and target VEGF receptor 1,2 and 3 (VEGFR 1,2,3) (31, 32). Sunitinib proved to be a promising first line therapy for metastatic renal cell carcinoma (mRCC) patients and, when compared to previously used standard-of-care treatment with interferon alpha (IFNα), it provided longer median survival and objective response rates (32). Thus, it was demonstrated that targeting tumor vasculature was a viable clinical option.

However, although providing promising results, it was shown that these therapies were not fully successful in eradicating tumor disease or in preventing its recurrence. It was concluded that the complexity of angiogenesis and various compensatory mechanisms that are involved in the regulation of this important process are responsible for this feature. Since antiangiogenic agents commonly target a single epitope of a particular molecule, due to compensation mechanisms, the process of angiogenesis is only temporarily affected. This results in a temporary inhibition of this process, which is quickly overcome, whereas resistance towards this type of therapy can be established (33). Additionally, some serious side effects due to non-specific targeting, involving but not restricted to hemorrhage, gastrointestinal perforation, and various complications with wound-healing and reproductive process, were attributed to application of targeted therapies, including bevacizumab (34–36).

**Figure 3:**
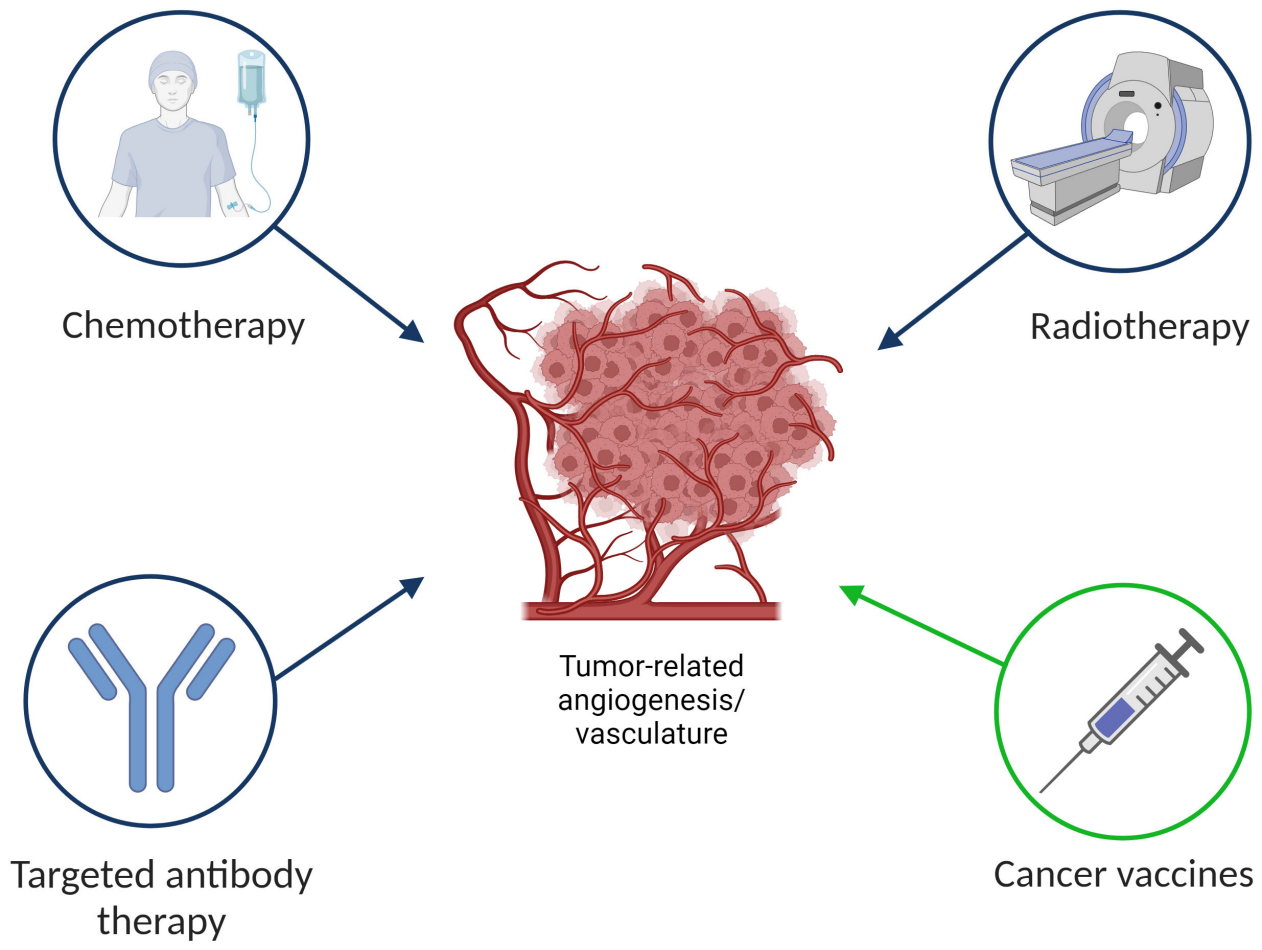
Standard of care anti-cancer therapies and anti-angiogenic vaccines.

Chemo- and radio- therapy are standard therapies used in cancer treatment and they also target the tumor vasculature (37–39). These therapies provide a certain success in cancer treatment depending on the histology and developmental phase of the cancer at hand and thus are considered conventional therapies (40). However, the presence of hypoxia as a cause of angiogenesis promotion has been linked with a bad prognosis and failure of different anti-cancer therapeutics including radio- and chemo- therapy (41). For instance, it was observed that low oxygen levels have detrimental effects on the response to ionizing radiation, thus preventing or lowering its effectiveness (42). On another example, HIF upregulation diminishes or limits effectiveness of chemotherapy through up-regulation of the adenosine triphosphate (ATP)-dependent drug efflux pumps (43) (**Figure 3**).

With all previously said, it can be concluded that to induce an efficient anti-angiogenic response we should broaden our perspective and try to target more than one epitope and one therapeutic target. In addition, this process should be more precise to prevent side effects. Besides, therapies or their combination should aim to relieve the hypoxic and immune suppressive features of this process, and induce the so-called normalization of vasculature, which would provide conditions for successful drug delivery and infiltration of immune cells with anti-tumor activity, thus eventually resulting in better response to therapy. Cancer vaccines have a high potential to achieve this and circumvent previously mentioned obstacles of anti-angiogenic therapies.

## 3 Anti-angiogenic cancer vaccines

The advantages of vaccine therapy over standard anti-angiogenic treatments such as monoclonal antibodies are multiple. For instance, it has been suggested that vaccines can induce more effective and broader response; the administration of vaccines could be less frequent and more cost effective than biologics. As compared to monoclonal antibodies, vaccines can simultaneously target multiple epitopes and induce stronger and polyclonal response, which may be necessary to overcome tumor evasion mechanisms. Vaccines require to be administered less often than monoclonal antibodies, and this could reduce/prevent possible establishment of resistance (44).

Furthermore, precise and quick translation of the cancer vaccines, as has been demonstrated by the messenger ribonucleic acid (mRNA)-based COVID-19 platforms, is currently revolutionizing the biomedical industry and the personalized medicine field (45).

The idea that the immune system could play a vital role in tumor destruction is very old and is the foundation of immunotherapy (46). It is well known that the immune system is involved in the extremely complex process of interaction with tumor cells, where it plays a crucial role in the detection, control and eradication of tumor threats (47). Cancer development involves a Darwinian selection process, and the presence of a tumor implies that the immune system failed in the timely eradication of that neoplastic formation. Nevertheless, a reactivation of the immune response may result in cancer eradication (48). Different immunotherapeutic modalities exist and include immunomodulatory antibodies (monoclonal antibodies and immune checkpoint inhibitors, ICIs), non-specific immunotherapy (cytokines etc.), viral oncolytic therapy, cellular immunotherapy (CAR T cells, NK cells, etc.), and cancer vaccines (49–51).

Cancer vaccines represent one of the most promising immunotherapeutic approaches against cancer and are based on the activation and/or re-activation of immune response against tumors upon immunization with antigens that are considered foreign by the immune system. These could be either non-specific self-aberrant proteins called tumor associated antigens (TAAs) or specific tumor proteins, called neoantigens or tumor specific antigens (TSAs) (52). TAAs represent antigens that are over-expressed in tumors but can be also expressed in normal tissue. These include differentiation and carcinoembryonic proteins but also pro-angiogenic factors/receptors. These antigens feature low tumor specificity, low immunogenicity and high central immune tolerance (53). TAAs can be also viral antigens in case of tumors induced by viral infections and, in this case, they feature high tumor specificity and high immunogenicity (54). On the other hand, TSAs represent mutated proteins that are a product of tumor cell non-synonymous mutations and thus feature higher tumor specificity, higher immunogenicity and a weaker central immune tolerance than TAAs (55).

In theory, the ideal antigen for cancer vaccines should be solely expressed on cancer cells (not on healthy cells), able to activate efficient immune response, and be vital for cancer cell survival. Therefore, TSAs seem to be the best choice. However, various obstacles were observed in identifying an appropriate immunogenic TSA for optimizing vaccine design and delivery, depending on the cancer type and stage of the disease. Due to the recent progress in genomic sequencing, *in silico* modeling and bioinformatics, identification of potential TSAs improved drastically providing conditions for more precise and quicker identification and application of these antigens (56). This resulted in the development of a cancer vaccine that was able to significantly prolong survival in melanoma patients in a recent clinical study (57). Notwithstanding the tremendous progress that has been made in identifying these antigens and their translation to the clinic, their effectiveness is still limited. One of the potential reasons is that out of the many predicted neoantigens, only a few actually induce a strong immune response. Additionally, due to differences among individual patients, these antigens are highly restricted to specific patient sub-populations and therefore manufacturing these vaccines is still complex and expensive (58). Therefore, targeting TAAs and/or a combination of TAAs with TSAs still represents the most realistic solution for cancer vaccine development.

Several of the cancer vaccines that were developed to target TAAs have been successful in non-clinical models and some of them have reached clinical studies. Some examples of therapeutic cancer vaccines, approved by the United States Food and Drug Administration (FDA) include Sipuleucel T (Provenge), a cancer vaccine for the treatment of metastatic prostate cancer; Bacillus Camlette-Guerin (BCG) and Nadofaragene firadonevec (Adstiladrin) for early-stage bladder cancer; and Talimogene Laherparepvec (T-VEC; Imlygic) for the treatment of advanced melanoma (59–62). Examples of FDA-approved preventive cancer vaccines include Gardasil and Cervarix, which target the human papilloma virus (HPV) and are useful to fight HPV-induced cancers (63). Due to such promising results, this research field has been steadily growing in the past decade and many cancer vaccines are in non-clinical and clinical phases of development, and in constant progress.

However, there are still many obstacles that limit the efficiency of these vaccines that need to be overcome. These obstacles include, but are not limited to, low immunogenicity of TAAs, different evading systems available to tumor cells, and development of a tumor immunosuppressive environment (64, 65). Another potential reason for limited efficacy of these vehicles is the fact that the tumor cells, due to their genetic instability and various immune escape mechanisms, can adapt by down-regulating their TAAs or TSAs, thus abrogating the immune response (66).

Promising results in overcoming obstacles related to the use of TAAs were observed with cancer vaccines targeting pro-angiogenic factors and tumor vasculature. It is well known that the tumor vasculature and stroma are genetically more stable than the cancer cells themselves, thus resulting in more stable expression of different TAAs that could be potentially recognized by immune cells, and lower possibility for resistance (67). Beside their stability, pro-angiogenic antigens are overexpressed in tumor tissues while they are rarely present in adult normal tissues, thus potential side effects are considerably lower than for other TAAs (68). In addition, pro-angiogenic antigens are broadly present in different tumor types, induce potent immune responses, and can be combined with other therapies (69, 70). Due to the fact that many cancers share these vascular markers, an anti-angiogenic vaccine could target various tumor types and potentially diminish the need for anti-cancer personalized therapy, which is more expensive (21). Therefore, different cancer vaccines are being developed that target crucial regulators of angiogenesis and antigens present on tumor vasculature, aiming to induce infiltration of lymphocytes, restore immunosurveillance, and induce anti-tumor immune responses (71).

**Figure 4:**
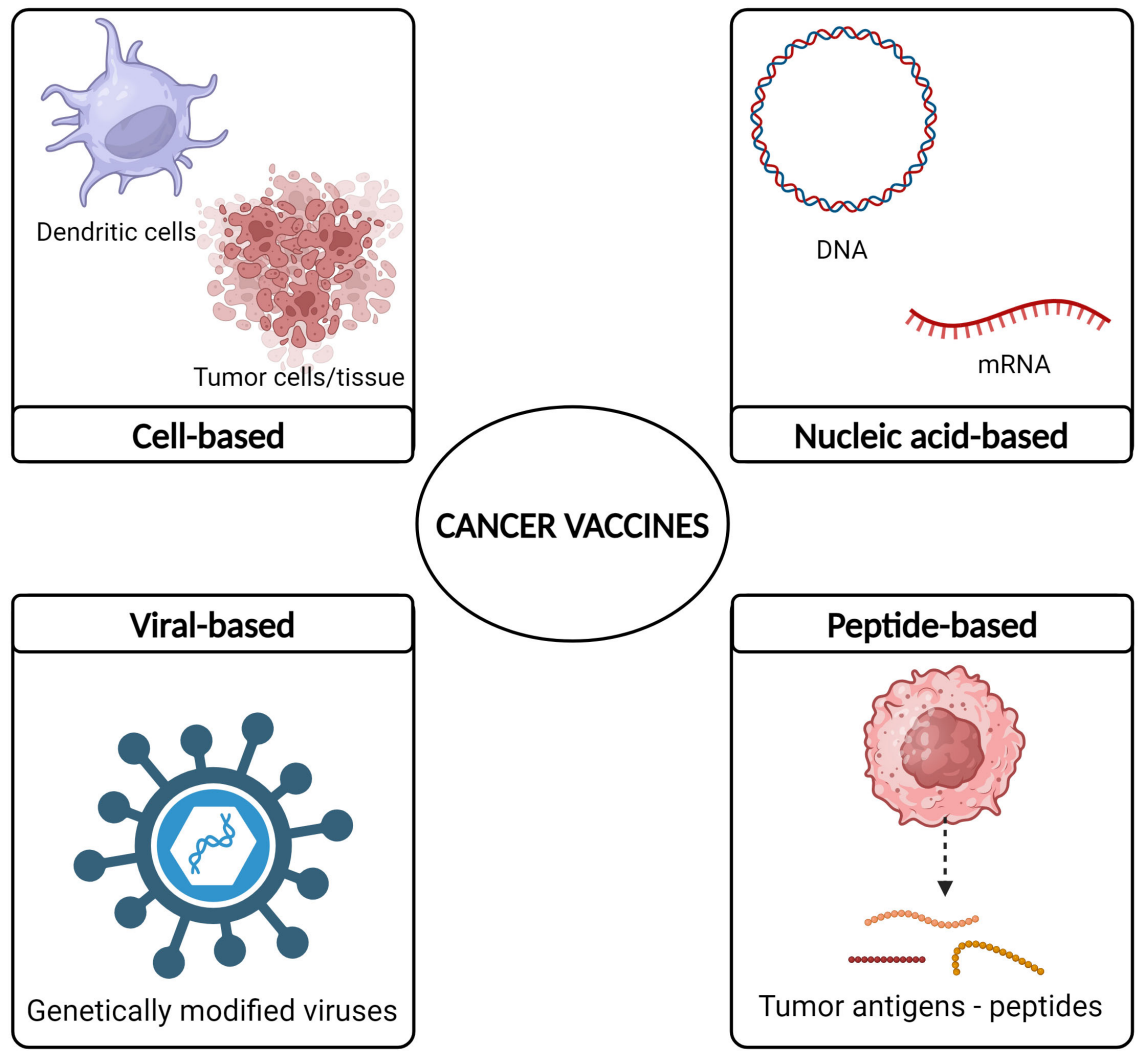
Cancer vaccine types.

Different cancer vaccine formulation have been developed and they include cell-based, viral-based, peptide-based, and nucleic acid-based vaccines (72–75) (**Figure 4**).

## 4 Nucleic acid vaccines against angiogenic markers

Some advantages of nucleic acid vaccines as compared to other types include their relatively simple design and production, low cost, the fact that they are easily modifiable and adaptable, and the possibility of encompassing multiple antigens simultaneously. In addition, these vaccines have the potential to overcome immunotolerance mechanisms, and have shown to be promising in anti-cancer and anti-angiogenic therapies (69, 76). Thus, these vaccines represent a feasible and promising anti-cancer therapeutic approach. Nucleic acid cancer vaccines include those designed with DNA or RNA backbones.

### 4.1 DNA cancer vaccines targeting tumor related angiogenesis (non-clinical studies)

**Figure 5:**
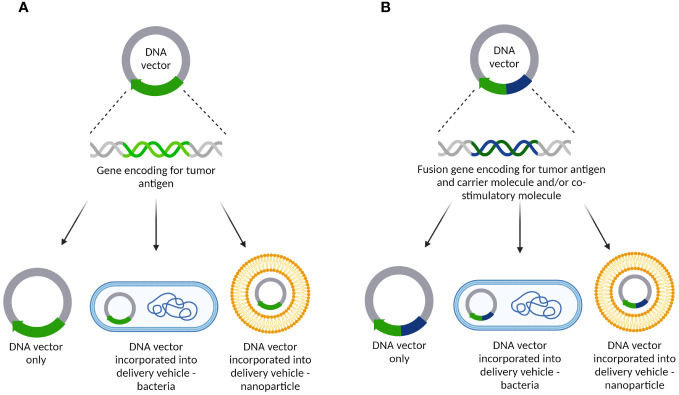
DNA cancer vaccine constructs targeting tumor-related angiogenesis/vasculature. **(A)** DNA vector containing a gene encoding for tumor antigens only. It can be delivered alone as a naked DNA vaccine or incorporated into delivery vehicles such as bacteria or different nanoparticles; **(B)** DNA vector carrying a fusion gene encoding for tumor antigen and carrier molecule and/or co-stimulatory molecule. As with the other plasmid, it can be delivered as a naked DNA vaccine or incorporated into delivery vehicles such as bacteria or different nanoparticles.

DNA cancer vaccines were developed during the 1990s targeting different TAAs and/or TSAs. They were based on the genetic sequence of a TAA or TSA that was incorporated into a bacterial plasmid either alone or bound to a hapten or carrier molecule such as *Tetanus Toxoid* (TTX), and/or a co-stimulating molecule (such as interleukin-2, IL-2), granulocyte-macrophage colony stimulating factor (GM-CSF), and/or others. Furthermore, the genetic construct could be inserted into a delivery vehicle, such as attenuated strains of *Salmonella typhimurium* or different nanoparticles (77) (**Figure 5**).

Various routes can be used to administer these vaccines and they include intramuscular (i.m.), intradermal (i.d.), subcutaneous (s.c.), mucosal, and oral. Since the DNA needs to be delivered to the cell nucleus to be able to induce an effect, and due to the difficulty of crossing cellular membranes, administration of DNA vaccines is usually followed by additional stimulation with electroporation, sonoporation, DNA tattooing or gene guns (78).

**Figure 6:**
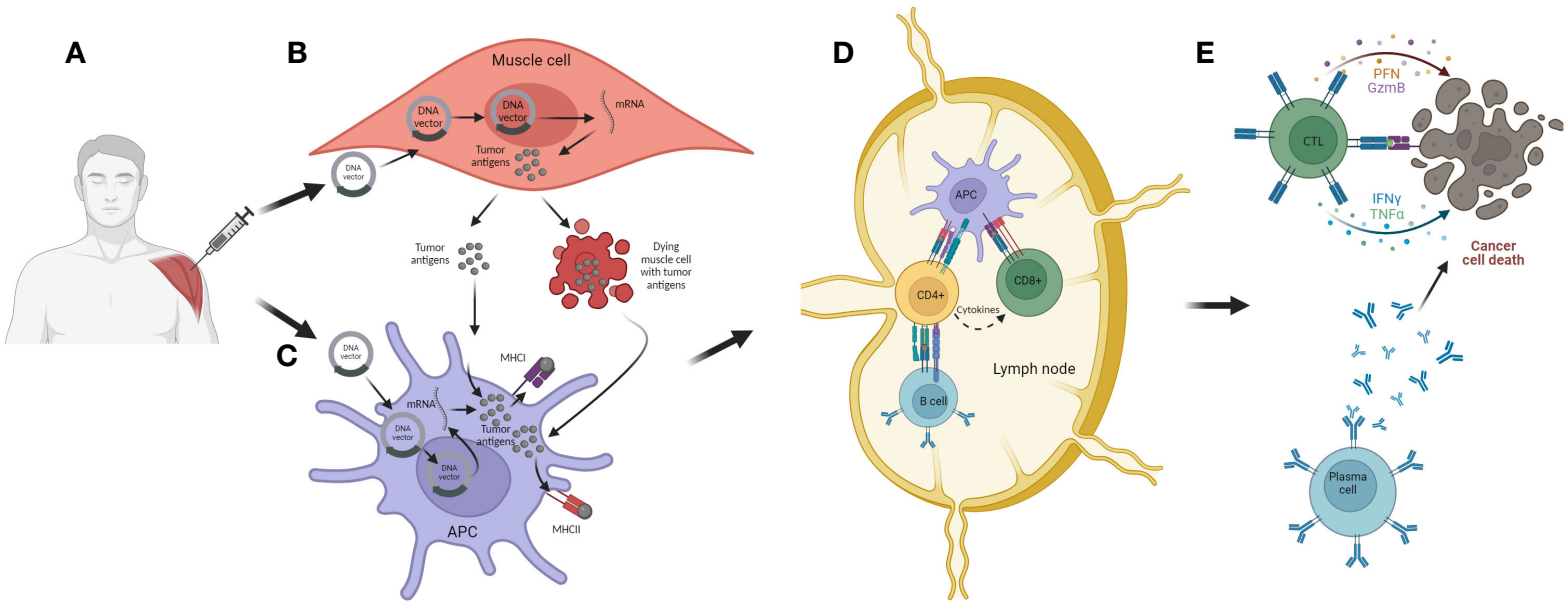
The mechanism of action of DNA cancer vaccines. **(A)** Intramuscular (i.m.) administration of the vaccine. **(B)** Transfection of muscle cells with DNA vector encoding for tumor antigen and/or carrier molecule and/or co-stimulatory molecules. Inside the nucleus, the DNA is transcribed into mRNA and exported to the cytosol where it is further translated into appropriate antigens. These are further processed and secreted as proteins or peptides into the extracellular environment. The transfected muscle cell can also undergo cell death as a consequence of mechanical damage during immunization and thus release tumor antigens; **(C)** APCs can be transfected with DNA vector but they can also uptake tumor antigens released from muscle cells. The tumor antigens are then processed and expressed together with MHC I and II. Activated APC then migrate to local lymph nodes where the antigen presentation process is accomplished; **(D)** APCs present tumor antigens to CD4^+^ and CD8^+^ T cells, thus activating these lymphocytes. Also, CD4^+^ T cell can activate B cells, which can also be activated with antigens released from APCs; **(E)** Activated lymphocytes migrate from the lymph nodes to the tumor microenvironment where, after recognition of target tumor cells, they are able to induce cell killing, either directly with cytotoxic granules released by CTLs or indirectly through antibodies generated from plasma cells.

The DNA could be delivered to antigen presenting cells or to regular cells, such as muscle cells, processed in the cell nucleus resulting in messenger ribonucleic acid (mRNA), which will be eventually translated into the desired tumor antigen. This protein then can be secreted to the extracellular environment and/or be further processed and presented in the cell membrane, associated to the major histocompatiblity complex (MHC) I and II. As a consequence of immunization and tissue damage caused by transfection, target cells can undergo apoptosis therefore releasing the antigens. Once dendritic cells (DCs) recognize and up-take these tumor antigens, they migrate to neighboring lymph nodes where they present tumor antigens to naïve CD4+ and CD8+ T cells (79). After presentation and co-stimulation, T cells are activated and trafficked to the tumor microenvironment (TME) where they recognize tumor cells expressing the antigen of interest and induce their killing (80). The CD4+ T cells are indirectly involved in tumor cell killing via several mechanisms. For instance, through activation of B cells and promotion of antibody-dependent cellular cytotoxicity (ADCC) (81). Additionally, the activated CD4+ T cells can actively secrete cytokines such as interferon y (IFN-y) and promote the expression of MHC I on tumor cells and also actively recruit different immune cells to the TME, thus promoting establishment of a pro-inflammatory environment aiming to support tumor eradication (82). Upon successful activation, CD8+ T cells differentiate into effector T cells, also known as cytotoxic T lymphocytes (CTLs), that after recognition of the cancer cells expressing the tumor antigen of interest, induce their killing, mainly through secretion of cytotoxic molecules such as perforin and granzymes, and/or by direct contact through Fas ligand (FasL)-mediated interactions (83). They can additionally release pro-inflammatory cytokines such as IFNγ and TNFα and further stimulate immune response. Beside inducing humoral and cellular anti-tumor immune response, these vaccines can potentiate the innate immune response against the double-stranded DNA which constitutes the backbone of the bacterial plasmid, thus aiding the anti-tumor eradication (84, 85) (**Figure 6**).

The first DNA cancer vaccines targeting tumor vasculature and angiogenesis factors were developed in the last decade of the past Century and the first non-clinical study was published in 2001 (86). In this report, Wei et al. designed and tested a DNA vaccine targeting VEGF based on a homologous xenogenic *Xenopus laevis* VEGF (XVEGF-p) as the antigen. They observed that the vaccine was able to successfully break the immune tolerance and induce an anti-tumor immune response against VEGF in both prophylactic and therapeutic murine models. Additionally, it was observed that this response was dependent of the incorporation of a xenogeneic sequence, thus confirming the importance of this approach for overcoming immune tolerance mechanisms against this TAA.

Following these promising results, various DNA vaccines were developed to target different pro-angiogenic factors and tumor vasculature markers in the last two decades. These targets can be generally classified into soluble pro-angiogenic factors such as VEGF (86); endothelial matrix markers such as MMPs (87, 88); endothelial cell membrane-associated targets such as angiomotin (89, 90), angiopoietin 1 receptor (TIE-2) (91), delta-like ligand 4 (DLL-4) (92), tumor endothelial marker 1 (TEM1) (93) and tumor endothelial marker 8 (TEM8) (94), and endoglin (95–97); or growth factor receptors such as fibroblast growth factor receptor (FGFR) (98), platelet-derived growth factor receptor β (PDGFRβ) (99), VEGFR-2 (100–108) and VEGFR-3 (109). In addition, several vaccines have targeted antigens expressed both on the tumor and endothelial cells and thus inducing a broader and enhanced immune response, for example targeting survivin (75) (110–123) (**Supplementary Table 1**).

#### 4.1.1 Endothelial matrix markers

Endothelial matrix markers are one of the targets exploited in some anti-angiogenic vaccine approaches considering their important role in endothelial cellular matrix degradation.

Yadav et al. (2020) engineered a DNA vaccine encoding a fusion of canine *MMP-7* and murine interleukin 18 (*Il-18*) gene sequences, comparing its efficacy with vaccines encoding *MMP-7* or *Il-18* alone (87). IL-18 is considered to be involved in promotion of pro-inflammatory cellular immune response mainly through up-regulation of IFN-γ and Fas ligand (FasL) on CD8+ T cells and natural killer cells (NK) cells, thus accomplishing FasL directed killing. In a murine mammary carcinoma model, the vaccine encoding *MMP-7* alone demonstrated considerable anti-tumor effects, characterized by reduced tumor growth, increased T cell infiltration, and prolonged survival. However, the fusion vaccine displayed superior efficacy, notably in reducing microvessel density, indicating the potential of IL-18 to enhance both humoral and cellular anti-tumor responses.

Su et al. (2003) developed a DNA vaccine targeting MMP-2, which significantly reduced tumor metastases and growth in mouse models (88). This vaccine formulation, comprising plasmid DNA encoding chicken *MMP-2*, induced a robust anti-tumor response in mouse models. Immunization with xenogeneic MMP-2 led to a significant reduction in tumor metastases in therapeutic models and inhibited tumor growth in prophylactic models, while the same was not observed with allogenic MMP-2. This vaccine elicited auto-antibodies against MMP-2, with CD4+ T cells identified as pivotal in driving the anti-tumor response. Remarkably, neither study reported any adverse effects, highlighting the safety profile of these anti-angiogenic vaccine strategies.

#### 4.1.2 Endothelial cell membrane associated targets

Angiomotin, serving as a receptor for the anti-angiogenic protein angiostatin, regulates endothelial cell migration and is a highly expressed angiogenic marker, making it a potential target. Holmgren et al. (2006) designed a DNA vaccine targeting human angiomotin and tested it in transplantable and transgenic mouse models (89). Immunization significantly impaired tumor growth and prolonged survival in transplantable tumor models, with CD4+ T cells and B cells identified as key mediators of the anti-tumor response. In the transgenic model the same was not observed. However, combining the angiomotin DNA vaccine with another targeting extracellular and transmembrane domains of human Her-2 resulted in dramatic tumor growth impairment in the transgenic model, demonstrating increased tumor-free survival. Moreover, the vaccine-induced antibodies prevented endothelial cell migration without adverse effects on physiological angiogenesis. In a follow-up study in 2012, the angiomotin DNA vaccine demonstrated efficacy in reducing tumor growth and increasing tumor-free survival in transgenic and transplantable tumor models (90). Additionally, it increased tumor vascular permeability, leading to epitope spreading and enhanced tumor regression when combined with doxorubicin. These findings suggest the potential of angiomotin-targeting DNA vaccines, alone or in combination therapies, for cancer treatment.

Ramage et al. (2004) devised a DNA vaccine targeting TIE-2, the angiopoietin-1 receptor, by incorporating modified *in silico* designed MHC I epitope of TIE-2 that is homologous for both mice and humans (91). By additionally modifying residues comprising epitopes of interest, authors were able to improve binding and specificity. They found that this vaccine stimulated a robust anti-tumor immune response in experimental settings, as demonstrated by the cytotoxicity of splenocytes against cells with high levels ofTIE-2 protein. Notably, no visible adverse effects were observed during the study, indicating potential safety of the vaccine. However, animals were not tumor challenged, thus further studies are necessary to confirm anti-tumor effect *in vivo*.

DLL-4 is a marker highly expressed during angiogenesis by endothelial tip cells and has been targeted for anti-angiogenic vaccination. In a study by Haller et al. (2010), a DNA vaccine against DLL-4 was developed using a pVAX1 expression vector containing human *DLL-4* cDNA (92). Immunization involved intradermal injection followed by *in vivo* electroporation. Mice immunized with the vaccine exhibited delayed tumor growth and prolonged survival compared to controls. The vaccine induced the production of anti-DLL-4 antibodies, which disrupted tumor vasculature without causing endothelial cell death, leading to non-productive tumor angiogenesis. This anti-tumor effect was attributed to humoral immunity against DLL-4.

Authors of all three studies did not report adverse effects thus suggesting that targeting of endothelial cell markers such as angiomotin, TIE-2 and DLL-4, in anti-angiogenic design offers a safe and promising avenue for cancer vaccine therapy.

#### 4.1.3 Tumor endothelial markers

Some DNA vaccine formulations target specific endothelial markers over-expressed in tumor-associated endothelial cells compared to normal ones, such as TEM-1 and 8.

Facciponte et al. (2014) targeted TEM-1/endosialin, a marker which is aberrantly expressed on tumor-associated endothelium and in tumor stroma, with a fusion DNA vaccine comprising mouse *Tem-1* cDNA attached to the C-terminal end region of *TTX* (93). Immunization significantly reduced tumor growth and delayed tumor onset in prophylactic settings across multiple tumor models. In therapeutic settings, immunization led to increased T cell infiltration in tumor stroma, indicating a cellular anti-tumor response mediated by T cells. Additionally, immunization induced apoptosis among tumor endothelial cells, leading to decreased proliferation and epitope spreading anti-tumor effects.

Ruan et al. (2009) developed a DNA vaccine targeting TEM-8, incorporating human *TEM-8* into a plasmid and using attenuated *S. typhimurium* as a carrier (94). In C57BL/6 mice, oral immunization significantly reduced tumor growth in a primary tumor model compared to controls. Immunized animals also developed fewer lung metastases in a metastatic tumor model. Splenocytes from immunized animals demonstrated cytotoxicity against TEM-8-expressing cells, mediated by CD8+ T cells.

These studies demonstrate the potential of DNA vaccines targeting TEM-1 and TEM-8 in eliciting robust anti-tumor responses without adverse effects, suggesting their promising role in cancer immunotherapy.

#### 4.1.4 Endoglin

Endoglin, another endothelial cell marker crucial for tumor growth regulation, has also been targeted for anti-angiogenic vaccination. Lee et al. (2005) developed an oral DNA vaccine against murine endoglin, delivered by *S. typhimurium* (95). In a prophylactic setting, immunization with this vaccine resulted in significantly fewer lung metastases and prolonged survival in mice challenged with murine breast carcinoma cells. Moreover, immunized animals exhibited up-regulation of activation markers on T cells and dendritic cells, suggesting a cellular immune response mediating the observed effects. Additionally, *in vitro* immune-mediated cytotoxicity assays showed that T cells induced specific lysis of cells expressing endoglin, thus confirming the efficacy and specificity of induced anti-tumor response.

Jarosz et al. (2013) expanded on these findings by testing a DNA vaccine against endoglin alone or in combination with interleukin-12 (IL-12) and cyclophosphamide (96). The vaccine alone was able to induce reductions in tumor size, prevention of recurrence, and decreased immunosuppression within the tumor microenvironment, without affecting wound healing. In addition, effects were confirmed *in vitro* with immune-mediated cytotoxicity assay. This combination therapy led to even more significant effects and still no side effects were observed, including wound healing.

Wood et al. (2011) took a different approach by developing DNA vaccines against murine endoglin fused with adjuvant sequences and delivered via an attenuated strain of *Listeria monocytogenes* (97). Testing in therapeutic settings across multiple murine breast tumor models demonstrated substantial reductions in tumor size, delayed tumor onset, and fewer metastases compared to controls. *In vitro* studies confirmed the secretion of IFNγ by splenocytes in response to tumor cell challenges, further supporting the anti-tumor efficacy of the vaccines. Additionally, the vaccines were effective in suppressing tumor neovascularization, as evidenced by reduced expression of vascular endothelial proteins and decreased tumor perfusion.

Together, these studies underscore the potential of anti-angiogenic DNA vaccines targeting endothelial cell markers for cancer therapy, offering promising avenues for further research and clinical development. However, the safety profile of these vaccines has not been investigated, or the information was not disclosed by authors, except by Jarosz et al., thus some precaution should remain in this aspect.

#### 4.1.5 Growth factor receptors

The growth factor receptors represent an important target due to their role in the angiogenesis process, thus multiple DNA cancer vaccines were developed to target them.

##### 4.1.5.1 FGFR-1

An important growth receptor is FGFR that mediates binding of FGF, one of the most abundant pro-angiogenic molecules involved in vital functions of endothelial and tumor cells. He et al. (2003) developed a xenogeneic DNA vaccine targeting mouse FGFR-1 by fusing mouse and frog FGFR-1 sequences (98). Tested in fibrosarcoma, hepatoma, and mammary gland tumor models in BALB/c mice, the vaccine provided significant protection against tumor growth in both prophylactic and therapeutic settings. Detection of auto-antibodies against FGFR-1 in immunized mice indicated an immune response. Depletion of CD4+ T cells abolished the anti-tumor response, while CD8+ T cell depletion had no effect, emphasizing the role of CD4+ T cells. Adverse effects, including delayed wound healing, were observed, thus suggesting that, although promising, FGFR-targeted DNA vaccines for anti-angiogenic therapy need to be further investigated in order to reduce and prevent side effects occurrence.

##### 4.1.5.2 PDGFRβ

One of the growth factors expressed in the tumor stroma is PDGFRβ. This receptor belongs to the tyrosine kinase receptor family and is over-expressed in several tumor stromal cells, such as pericytes and tumor fibroblasts. In Kaplan et al.’s study (2006), murine platelet-derived growth factor receptor β (PDGFRβ) was targeted with a DNA vaccine delivered by attenuated *S. typhimurium* (99). Tested in both preventive and therapeutic settings across murine colon, lung, and breast cancer models, the vaccine induced cell-specific lysis of PDGFRβ-expressing tumor cells without affecting non-expressing cells. It effectively inhibited tumor growth and metastasis in all models. Furthermore, vaccination reduced PDGFRβ levels and expression of the pericyte-specific marker NG-2 in tumors. Matrigel assays showed significant suppression of angiogenesis. Notably, no adverse effects were reported, indicating the vaccine’s potential for anti-angiogenic therapy with no apparent toxicity.

##### 4.1.5.3 VEGFR-2

VEGFR-2 is the main receptor of VEGF on the surface of endothelial vascular cells and it is central for angiogenic signaling. Niethammer et al. (2002) developed a DNA vaccine using attenuated *S. typhimurium* to deliver a murine *Vegfr-2* construct orally (100). Their vaccine demonstrated promising results in both prophylactic and therapeutic settings, showing prolonged survival and reduced lung metastases in animals. It elicited a cytotoxic immune response mediated by CD8+ T cells and led to a significant reduction in tumor vessel density. The immunized animals had delayed wound healing but no adverse effects were found on fertility.

Liang et al. (2010) designed a fusion DNA vaccine by combining *Vegfr-2* with complement component 3d (C3d) and administered it intramuscularly (101). C3d promotes both innate and adaptive immune response to antigens. This vaccine induced high anti-VEGFR-2 antibody titers and exhibited potent anti-tumor effects, reducing tumor growth and increasing the lifespan of immunized animals after tumor challenge. The study highlighted the potential of complement components as effective adjuvants in DNA vaccine formulations targeting angiogenesis.

Wang et al. (2007) developed a DNA vaccine by fusing *Vegfr-2* with murine beta defensin 2 (MBD-2), molecule that is involved both in innate and adaptive immune response and is very important for recruitment and maturation of antigen presenting cells, thus constituting a potent adjuvant (102). The fusion DNA construct was encapsulated into cationic liposomes and delivered. This vaccine demonstrated significant reductions in tumor growth and lung metastases in both prophylactic and therapeutic settings. The immune response was mediated by CD4+ T cells, CD8+ T cells, and B cells, and the vaccine showed anti-angiogenic effects by reducing tumor vessel density.

Lu et al. (2008) combined oral and intratumoral DNA vaccines targeting murine VEGFR-2 and murine interferon-induced protein-10 (IP-10) (103). IP-10 serves as a pro-inflammatory chemokine and promotes formation of pro-inflammatory environment thus acting as an adjuvant. *S. typhimurium* was used as a carrier for orally delivered DNA vaccines against murine VEGFR-2. This combination resulted in synergistic effects, with enhanced reductions in tumor size, tumor vessel density and increased survival compared to individual vaccines. Combination of vaccines outperformed all the single vaccines, thus suggesting that the combination of different DNA vaccine targets could improve therapeutic outcomes.

Another approach utilized live attenuated *S. typhimurium* coated with self-assembled nanoparticles made from cationic polymers carrying a VEGFR-2 vector (104). Mice immunized with this vaccine showed elevated levels of CD4+ and CD8+ T cells, along with increased pro-inflammatory cytokines, such as tumor necrosis factor alpha (TNFα), IFNγ and IL-12, suggesting that both humoral and cellular immune response were induced. This resulted in reduced tumor growth and vascularization as well as in improved survival rates, indicating a robust anti-tumor immune response induced by the vaccine. Besides, this study confirmed the efficacy of nanoparticles when used as delivery vehicles for these vaccines.

Wen et al. (2016) developed a fusion DNA vaccine combining human *VEGFR-2* and *IL-12*, administered via i.m. followed by electroporation (105). Their vaccine exhibited superior tumor growth inhibition compared to DNA vaccine with VEGFR-2 alone, without any reported side effects. The study suggested the potential of combining VEGFR-2 with immune-stimulatory molecules such as IL-12 for enhanced anti-tumor effects.

In the 2016 study by Gao et al, authors tested a fusion DNA cancer vaccine targeting the E6E7 motifs from human papillomavirus (HPV) and human VEGFR-2 (106). Animals were immunized i.m. followed by electroporation and tested in a therapeutic setting in C57BL/6 mice bearing lung tumor cells expressing E6 and E7 (TC-1 E6E7). Antibody titers induced by the fusion vaccine were similar to those obtained with a vaccine targeting E6E7 alone. Nevertheless, IFNγ levels were higher in animals immunized with the fusion vaccine, suggesting induction of a strong Th1 response by the VEGFR-2 moiety. The fusion vaccine displayed also a better protection against tumor growth. Authors did not report side effects.

Denies et al. conducted several studies evaluating DNA vaccines targeting human VEGFR-2. In the first study, the vaccine was tested in lung and breast transplantable mouse tumor models (107). While prophylactic benefits in terms of reduction in tumor size and prolonged survival were observed, therapeutic efficacy varied across different tumor models. Interestingly, in some models, immunization against VEGFR-2 promoted the development of liver metastases, indicating the complex interplay between immune response and tumor progression. Additionally, authors combined immunization with surgical removal of tumor but the vaccine was not effective in terms of tumor recurrence prevention.

In a second study, Denies et al. investigated the immunogenicity and safety of a DNA vaccine targeting human VEGFR-2 in mice and dogs (108). The vaccine was administered i.d. followed by electroporation. It induced significant tumor specific cellular and humoral responses in both species without any adverse effects observed, suggesting its potential for further clinical development. Interestingly, after a single injection in mice, the cellular response was significantly elevated whereas the humoral response was induced after two additional boosters. Conversely, in dogs both cellular and humoral response were significantly increased after the 3rd immunization suggesting differences in immune response among species that should be taken into account in translational research.

The above-mentioned studies support the use of VEGFR-2 as a promising and highly safe strategy in the anti-angiogenic cancer vaccine approach.

##### 4.1.5.4 VEGFR-3

Chen et al. (2016) developed a DNA cancer vaccine against murine VEGFR-3 targeting angiogenesis in lung cancer (109). VEGFR-3 is an endothelial marker but is also expressed in some cancer cells. VEGFR-3 is implicated in both angiogenesis and lymphangiogenesis, and its expression is correlated with bad prognosis in lung cancer bearing patients. Administered orally via attenuated *S. typhimurium*, the vaccine induced significant humoral and cellular immune responses in prophylactic and therapeutic models. Elevated levels of Th1-related cytokines were observed. Immunization led to reduced tumor growth, longer median survival, and decreased tumor lymphatic vessel density, suggesting dual anti-angiogenic and anti-lymphangiogenic effects. However, the study did not assess potential side effects associated with the administration of the DNA vaccine. While the results demonstrate promising anti-tumor efficacy, further research is needed to evaluate the safety profile of the vaccine, particularly in clinical settings.

#### 4.1.6 Markers expressed on both tumor and endothelial cells

Survivin is a marker highly expressed both in cancer cells and endothelial cells during angiogenesis. Survivin inhibits apoptosis, promoting the survival of tumor and endothelial cells, making it an attractive target for anti-angiogenic therapy.

In 2005, Xiang et al. developed a DNA vaccine targeting a fusion product of mouse survivin and C-C chemokine motif 21 ligand (CCL-21), a chemokine that attracts antigen-presenting cells and naïve T cells while having angiostatic ability, thus enhancing the immune response (110). The vaccine was delivered via attenuated *S. typhimurium*, a carrier known for its ability to deliver DNA vaccines to immune cells efficiently. This study demonstrated the effectiveness of the survivin-CCL-21 DNA vaccine in inhibiting tumor growth and inducing apoptosis in a murine model. Besides, no negative effects affecting either wound healing or fertility of immunized animals were found.

Zhang et al. conducted a study, in 2014, where they modified a DNA-based replicon vaccine derived from the Semliki Forest virus (SFV) to target human survivin and/or human chorionic gonadotropin beta core fragment (hCGb-CTP37) (111). The vaccine also included co-stimulatory molecules such as B7.1, immunoglobulin G Fc fragment (IgGFc), and the adjuvant GM-CSF. This chimeric construct induced successful humoral and cellular responses and significantly reduced tumor growth in both prophylactic and therapeutic settings, without observed adverse effects. Furthermore, combining this vaccine with another one targeting human VEGFR-2 and IL-12 showed synergistic anti-tumor effects, highlighting the importance of multi-targeting approaches in cancer immunotherapy (75).

Liu et al. employed a different approach by fusing the human survivin sequence with the human *MUC-1* sequence and inserting it into a bicistronic vector containing human IL-2 (112). This construct was further improved by incorporating an unmethylated cytosine-guanine dinucleotide (CpG) motif as an adjuvant. Their research demonstrated that this vaccine, either as a bicistronic expression vector or as two separate vectors, significantly reduced the number and size of metastases, prolonged animal survival, and modulated the tumor microenvironment by up-regulating pro-inflammatory molecules and down-regulating tumor-promoting factors.

The same group tested the slightly modified version of this vaccine where they added the sequence of soluble PD-1 (sPD-1) aiming to improve delivery of antigens to dendritic cells, thus improving activation and proliferation of T cells (113). The vaccine was tested in a therapeutic setting in mouse models and was able to induce both humoral and cellular immune response and significantly impair tumor growth. Additionally, maturation markers of DCs and T cells were up-regulated thus suggesting successful implementation of sPD-1. However, use of sPD-1 should be carefully considered given its potential immune-suppressive effect. In both studies, authors combined the vaccine with oxaliplatin and observed significantly improved anti-tumor effects but also enhanced toxicity. The modified bicistronic vector approach, having the same efficacy, can be a cheaper alternative as compared to separate vaccines.

Human survivin was used in another study where it was fused to human fibroblast activating protein alpha (FAPα) in a vector containing a CpG motif (114). FAPα is a marker of cancer associated fibroblasts (CAFs), which play an important role in the regulation of tumor microenvironment (TME) and in tumor evasion mechanisms. The immunization was performed with or without previous chemotherapeutic treatment with doxorubicin aiming to remove peripheral MDSCs. The fusion DNA vaccine was able to impair tumor growth and reduce the weight and size of the tumors while significantly prolonging median survival of animals in both models. Additionally, the fusion vaccine increased infiltration of the tumor by CTLs, while reducing the number of FAPα+ CAFS and MDSCs. Pro-inflammatory cytokines such as IFNγ and TNF-α were elevated while anti-inflammatory cytokines such as IL-10 were down-regulated, thus confirming the effect of the vaccine on regulating the TME. Authors did not disclose any information about potential side effects.

In another study, human survivin was targeted with a minigene DNA cancer vaccine (115). In their approach, an *in silico* prediction system was used to find parts of the survivin sequence with high immunogenicity and high affinity towards MHC I (H2-Kk). This approach aimed to reduce/prevent potential side effects of full length survivin gene insertion into tumor cells that could lead to prevention of apoptosis and thus tumor survival. Administered orally via attenuated *S. typhimurium*, the vaccine significantly impaired tumor growth and reduced liver metastases in a neuroblastoma model. The minigene vaccine induced a cellular immune response dominated by CD8+ T cells. In the therapeutic setting, it achieved complete tumor eradication in half of the animals and impaired tumor growth upon re-challenge in 80%, with no observed side effects, including wound healing, indicating its safety and potential efficacy.

In 2008, Peng et al. developed a DNA cancer vaccine encoding a phosphorylation-defective mouse threonine 34 to alanine mutant survivin (T34-A) (116). This mutant survivin had reduced anti-apoptotic function, preventing pro-tumoral effects. Liposomal encapsulation facilitated intravenous delivery of the vaccine. In therapeutic settings using a BALB/c mammary tumor model, animals immunized with this vaccine exhibited impaired tumor growth, reduced lung metastatic nodules, decreased microvessel density, and increased apoptotic endothelial cells. Additionally, the vaccine-associated splenocytes demonstrated cytolytic activity against tumor cells, indicating a robust anti-tumor immune response. No obvious side effects or toxicities were observed, although wound healing and reproduction tests were not reported.

In 2010, Yu et al. tested the T34-A mutant murine survivin DNA vaccine in combination with cisplatin chemotherapy in a murine Lewis lung carcinoma model (117). The combination therapy suppressed tumor growth and reduced microvessel density, with the vaccine demonstrating superior effects compared to chemotherapy alone on vessel density reduction. Similarly, Yuan et al. investigated the T34-A mutant survivin DNA vaccine in combination with radiotherapy in a murine lung carcinoma model, observing enhanced anti-tumor responses with increased apoptosis of tumor cells and decreased microvessel density compared to individual therapies (118). These results suggest an enhanced tumor cell sensibility to radiotherapy as a consequence of immunization.

Qiu and Zhao evaluated the T34-A mutant murine survivin DNA vaccine in a therapeutic setting using a murine cervical cancer model (119). Immunization with this vaccine resulted in reduced tumor growth, fewer tumor nodules, and decreased ascites fluid volume. Additionally, immunized animals exhibited reduced expression of platelet endothelial cell adhesion molecule 1 (PECAM-1) in tumor tissues compared to controls, indicating decreased angiogenesis and tumor progression. In 2010, Pan et al. investigated the T34-A mutant survivin DNA vaccine in a murine prostate tumor model (120). Administration of the vaccine led to impaired primary tumor growth, decreased microvessel density, increased tumor cell apoptosis, and reduced tumor vascularization. No evident cytotoxicity or organ damage was observed, highlighting the safety profile of the vaccine.

Wang et al. employed a combination approach involving two plasmids, one encoding a fusion gene product of human survivin and human chorionic gonadotropin (*hCG*), and the other coding for *IL-12* (121). In a murine breast carcinoma model, this combination vaccine demonstrated significant impairment of tumor growth, with superior anti-tumor effects observed compared to individual vaccines. Importantly, no adverse effects were reported, suggesting the safety and efficacy of this combinatorial approach.

Liadser et al. further explored survivin-targeted DNA vaccines in 2006 and 2010. In the 2006 study, they tested naked DNA vaccines encoding for non-secreted and secreted human survivin in BALB/c mice, delivered intramuscularly and followed with additional injection of cDNA encoding for murine GM-CSF serving as an adjuvant (122). Both constructs induced robust humoral and cellular immune responses against survivin, with the secreted form demonstrating greater potency. The cellular response was characterized by CD8 IFNy-positive T cells, while the humoral response was mainly Th1 CD4 directed. In the 2010 study, a DNA vaccine encoding an *in silico* predicted CD8+ T cell epitope of human survivin was evaluated (123). This vaccine successfully induced CD8 IFNy-positive cell immune responses and blocked tumor-related angiogenesis, providing significant protection from tumor development in a prophylactic and therapeutic setting. Additionally, in a prophylactic setting, tumor regression was observed and animals remained tumor-free. No information about assessment of side effects were disclosed.

In summary, the studies with DNA vaccines targeting surviving have demonstrated high efficacy and safety, together with a high potential for the strategy of targeting markers present on both tumor and endothelial cells.

### 4.2 Clinical studies testing DNA cancer vaccines targeting tumor related angiogenesis

A single DNA cancer vaccine targeting an angiogenic factor (VXM01) has been tested in clinical settings (124–127) (**Supplementary Table 2**). This is a modification of the vaccine developed by Niethammer et al., 2002, directed against VEGFR-2, but substituting the mouse VEGFR2 by its human counterpart.

In a first phase I clinical trial, advanced pancreatic cancer patients were enrolled (NCT01486329) (124). Patients were vaccinated four times during a week and the vaccine, carried by *S. typhimurium*, was given orally. The vaccine was generally well accepted, and the few adverse effects were transient and involved decreased levels of lymphocytes and platelets. Before starting the immunizations, several patients had detectable levels of T cells with antibodies against VEGFR-2, which could be a sign of the spontaneous reaction of the immune system to expanding tumor vasculature at this advanced setting. As expected, immunization further increased the levels of these VEGFR-2 effector T cells. Following immunization, an increase in tumor-infiltrating T cells was reported. Interestingly, tumor perfusion was reduced in immunized patients and serum markers, including increased levels of VEGF-A and collagen IV, increased; indicating that angiogenesis was clearly targeted. Curiously, in patients with pre-existing high levels of VEGFR-2 specific T cells, immunization effects were longer lasting as compared to patients with lower pre-existing levels. Furthermore, in the group with low pre-existing levels of VEGFR-2 specific T cells, Treg cell levels increased after immunization, thus promoting a tolerogenic state in the TME. Being a phase I clinical trial, no differences in clinical outcome were found, but remarkable changes were observed which are compatible with the induction of a strong immunosuppressive environment for some patients. Later on, in an extended phase I trial, additional booster doses were tested, 6 times separated by monthly intervals (125). Beside the placebo control group, two vaccine doses (high and low) were tested. Similar side effects and results (transient increases in specific effector T cells levels) were observed as compared with the first trial.

The same vaccine was then tested in a phase I/II clinical trial in patients suffering from progressive operable glioblastoma (NCT02718443) (126). The study involved 14 patients, 3 of which received an additional immune checkpoint inhibitor (nivolumab). The vaccine was administered 4 times during the first week, and then once every 4 weeks up to 48 weeks. Immunization resulted in increased infiltration of CD8+ T cells into the tumors and a higher CD8+ T cell to Treg ratio correlated with patient survival. Furthermore, one complete response was observed, and a favorable outcome was obtained in 5 out of 14 patients. Most of the side effects reported were not related to the immunization.

The same vaccine was also tested in combination with another immune checkpoint inhibitor (avelumab), in a larger cohort of 30 glioblastoma patients in a phase I/II clinical trial (NCT03750071) (127). Patients were previously treated with radiotherapy and the immunization schedule was identical to the previous study. VEGFR-2 T-cell specific IFNγ responses were observed in several patients. In three patients, a partial response was reported. As in the previous trial, the positive correlation between higher CD8+ T cell to Treg ratio correlated with patients´ survival and immune response. No major toxicities were found.

By reviewing the state of the art of DNA cancer vaccines targeting angiogenic factors, it can be concluded that these vaccines were able to break immune tolerance mechanisms and induce potent immune responses against their TAAs. Furthermore, the response was sufficient to prevent or impair tumor development and metastasis in non-clinical studies. In clinical settings, it was observed that elevation of antigen specific effector T cells was achieved when DNA cancer vaccines targeting angiogenic factors were used alone. However, the effect was not sufficient to abrogate tumor development, suggesting that combination with other therapies is necessary. On the other hand, we must take into consideration that these vaccines were tested in patients with advanced stages of the disease. In this setting, it could be difficult to induce an effective immune response against the tumors considering the strong immune-suppressive environment, exhaustion of T cells, and many other features that characterize the TME in such advanced stages.

Additionally, it could be concluded that DNA cancer vaccine strategy impose certain challenges or limitations that can affect complexity, efficacy and eventually safety of these vehicles. For instance, DNA needs to be delivered to the cell nucleus, transcribed to mRNA of interest which then needs to be delivered to the cytosol, where it can be translated into the antigens of interest. This means that two membranes, and all mechanisms controlling transfer through these membranes, need to be overcome so that the DNA can be delivered. Although electroporation was successfully used in non-clinical settings as a transfection method, it is not as feasible in clinical settings. The transcription to mRNA and export to cytosol represent additional obstacles and make this approach more complex and prone to possible failure due to intrinsic mechanisms. Therefore, this could further lead to reduced translation efficiency and lower immunogenicity. Furthermore, the possibility of DNA incorporation into the genome, although mostly theoretical, still needs to be considered when utilizing these constructs.

After reviewing the contributions of DNA technology to anti-angiogenic vaccines, we turn now to vaccines based on RNA approaches. The use of mRNA to prepare cancer vaccines could provide additional features and offer solutions to some of the discussed challenges affecting DNA vaccines, thus potentially resulting in more efficient anti-tumor immune responses. These RNA vaccines represent highly promising strategies that can be used to target tumor related angiogenesis.

### 4.3 mRNA cancer vaccines

RNA vaccines are using the mRNA molecule to provide instructions to the ribosomes that are present in the cell cytoplasm for generating the desired proteins (antigens) (128). The first reports of successful *in vivo* transfection of *in vitro* translated (IVT) mRNA was reported in 1990 by Wolf et al. (129). Subsequently, in 1992, Jirikowski et al. successfully used a mRNA vaccine encoding vasopressin to reverse diabetes in rats (130). These studies prompted further development of mRNA vaccines for various infectious diseases, but also for cancer. The first proof-of-concept demonstrating that cancer mRNA vaccines could be used for cancer treatment was published in 1995 by Conry et al., where they used a naked mRNA vaccine targeting human carcinoembrionic antigen (CEA) and were able to detect anti-CEA specific antibodies in immunized animals (131).

Various studies followed, but soon it became evident that early mRNA vaccine formulations were unstable, they induced high innate immunogenicity, and could not be successfully delivered to target tissues and cells. Therefore, research was diverted towards protein- and DNA-based vaccine development (132–134). After surmounting a lot of hurdles, mRNA technology has been drastically improved and, in 2020, it resulted in an FDA emergency approval for the use of two mRNA vaccines, Pfizer BioNTech/BNT162b2 and Moderna/mRNA-1273, against severe acute respiratory syndrome coronavirus 2 (SARS-CoV-2) (135, 136). Furthermore, the 2023 Nobel prize in Physiology or Medicine was awarded to pioneers of mRNA vaccine technology Dr. Katalin Karikó and Dr. Drew Weissman (137). Although no cancer mRNA vaccine has yet been approved by the FDA, currently there are 76 clinical trials involving mRNA cancer vaccines targeting solid tumors. From this number, 9 are not yet recruiting, 18 are recruiting, 27 completed, 8 terminated, 2 withdrawn, 5 with unknown status, 1 suspended and 6 active but not yet recruiting [search terms used: cancer; mRNA cancer vaccine; ClinicalTrials database accessed on (7th of May2024)] (138).

Among the many advantages that mRNA vaccines may have over previous technologies, we could report ease of fabrication, low costs, high safety and efficacy, but also ease of adaptation to new targets, and multi-targeting (139). The core of such vaccines is a synthetic mRNA molecule generated as a product of an *in vitro* transcription reaction (IVT), in which a phage polymerase such as T3, T7 or Sp6, synthesize mRNA from a linear DNA template in the presence of nucleoside triphosphates (NTPs). Following this, the DNA template is degraded and the mRNA capped so it closely resembles the naturally occurring mRNA molecules in the eukaryotic cells and can be translated efficiently (140). The synthetic mRNA should contain the open reading frame (ORF) which encodes for the gene of interest (GOI), 5’ and 3’ untranslated regions (UTRs), 5’cap, and polyadenylic acid tail (poly A tail). Each of these structures has a distinctive role and is essential for the stability and translation efficiency of the mRNA (141).

The ORF structure represents the sequence for the protein of interest. Optimization of the coding sequence can result in improved translational efficacy. Furthermore, addition of signaling tags can determine the fate of the antigen, generating either membrane bound or secreted immunogens, or can further specify its function, for instance by fusing the antigen to histocompatibility complexes MHCI or MHCII (134).

On the other hand, UTRs, 5´-cap and poly-A tail are functional non-coding parts of mRNA molecule crucial for its maturation, translation but also stability and timely-removal, thus modifying these structures can lead to improved functionality of mRNA based therapeutics (142–145).

**Figure 7:**
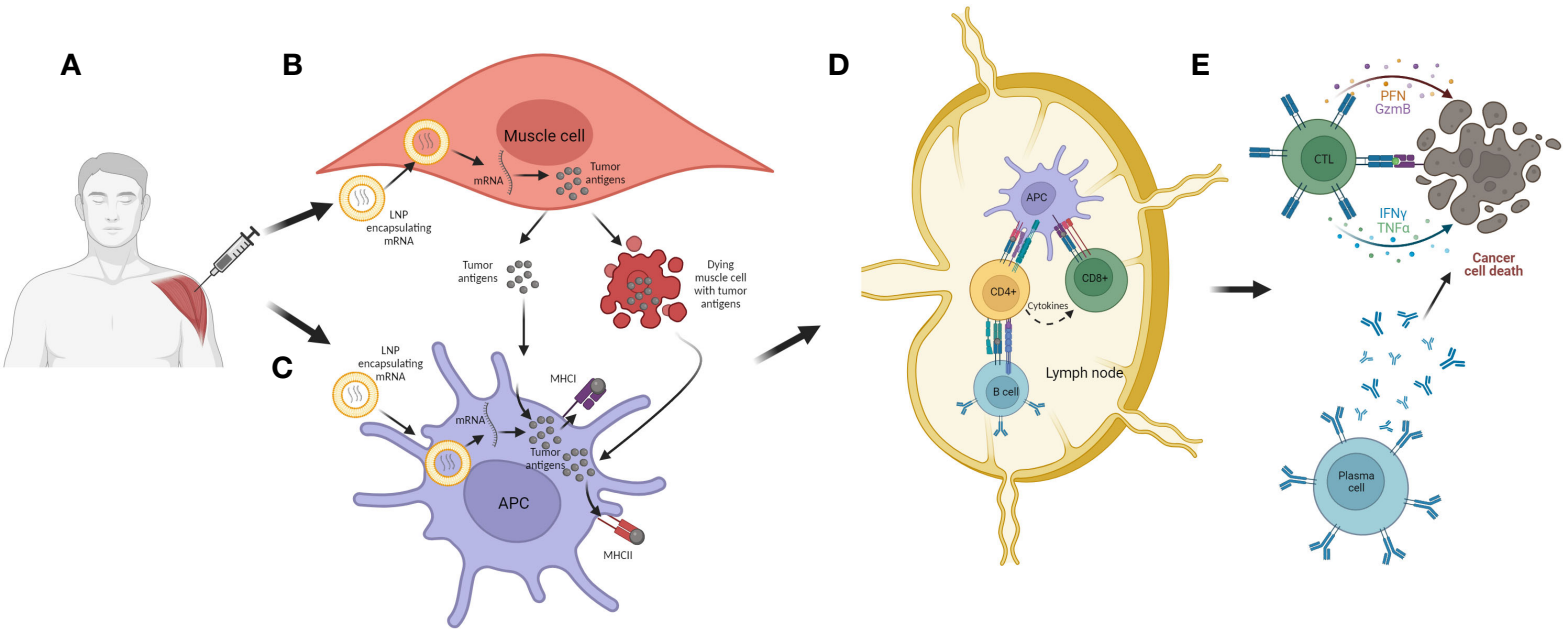
The mechanism of action of mRNA cancer vaccines. **(A)** Administration of vaccine (i.m.). **(B)** Transfection of muscle cells with mRNA encoding for tumor antigen encapsulated in LNPs. The mRNA is then translated into the tumor antigens in cell cytoplasm, and these are further processed and secreted as proteins or peptides into the extracellular environment. The muscle cells can also undergo cell death as a consequence of mechanical damage during immunization and thus release tumor antigens; **(C)** APCs are transfected with mRNA encoding for tumor antigen encapsulated in LNPs. They can also uptake tumor antigens released from muscle cells. The tumor antigens are then processed and expressed together with MHC I and II, and the APCs can migrate to local lymph nodes or the spleen; **(D)** APCs present tumor antigens to CD4^+^ and CD8^+^ T cells, and in that way they activate these lymphocytes. Also, CD4^+^ T cells can activate B cells which can also be activated with antigens released from APCs; **(E)** Activated lymphocytes migrate from the lymph nodes to the tumor where, after recognition of target cells, they are able to induce cell killing, either directly with cytotoxic granules released by CTLs or indirectly with antibodies generated by plasma cells.

As described on **Figure 7**, the mechanism of action of mRNA cancer vaccines is similar to DNA cancer vaccines, with a few differential steps. Initially, the mRNA administered either alone (naked) or encapsulated/conjugated to delivery vehicles, such as lipid nanoparticles (LNPs), is up-taken by APCs and muscle cells mostly through endocytosis. Then, the mRNA escapes from the endosomes and passes to the cytosol, where it is translated to peptides/proteins of interest that, in turn, will be secreted or presented together with MHC I and/or II. The final steps are the same as in the case of DNA cancer vaccines (**Figure 7**).

Exogenous mRNAs usually trigger a cellular defensive response since they can be associated with viral structures. When a foreign mRNA enters a cell, it is recognized as a pathogen associated molecular pattern (PAMP) by pattern recognition receptors (PRRs) which results in mRNA destruction and thus in translation inhibition. Furthermore, this process abrogates the initiation of the adaptive immune response, which is necessary for establishment of the immune memory against the antigen of interest (146). To reduce this problem, two solutions have been developed: first, removing the double stranded RNA, which is a co-product of IVT reaction, through purification. This approach reduces cellular responses (147). Secondly, incorporation of non-natural or naturally-occurring modified nucleosides, which helps in preventing innate immune recognition and improves translatability of the mRNA. Most commonly, adenosine could be substituted by N6-methyladenosine (m6A), cytidine by 5-methylcytidine (m5C), and uridine by either 5-methyluridine (m5U), 2-thiouridine (s2U), or pseudouridine (ψ). In addition, these substitutions increase ribosome density and protein expression, further improving efficacy of these therapeutics (148).

The mRNA cancer vaccines can be administered through various routes including intramuscular, intravenous, intradermal, subcutaneous, intranasal and intranodal (149). The most common route for delivery of mRNA cancer vaccine is intramuscular, which is very easy and has beneficial immunological aspects. *In vivo* mRNA activity is extremely dependent on the delivery method and, therefore, different delivery approaches have been developed. mRNA vaccines can be delivered as naked, as *ex vivo* electroporated DCs, or encapsulated/conjugated to different nanoparticles of which the most common are LNPs (150–152). mRNA needs to be delivered to the cell cytoplasm to be successfully translated into protein by the cellular translation machinery. Uptake of the mRNA is cell-specific and the physicochemical properties of the mRNA are crucial for its success. One of the critical improvements for the clinical use of mRNA vaccines was the development of carrier structures such as lipid nanoparticles (LNPs) (153). LNPs are cationic carriers approved by the FDA as the most efficient delivery vehicles for IVT mRNA. These positively charged lipids are able to pack and condense negatively charged mRNA, thus preventing its degradation and enabling its delivery to target cells. Besides providing protection, these carriers promote cellular uptake by APCs, a crucial step for the initiation of an immune response cascade. Furthermore, although the mechanism of action is not fully understood, most likely these particles are up-taken by cells through endocytosis and their interaction with endosomal membrane lipids leads to endosomal membrane disruption resulting in endosomal escape and release of the entrapped mRNA into the cytosol, thus enabling its further processing and translation in the ribosomes (154).

**Figure 8:**
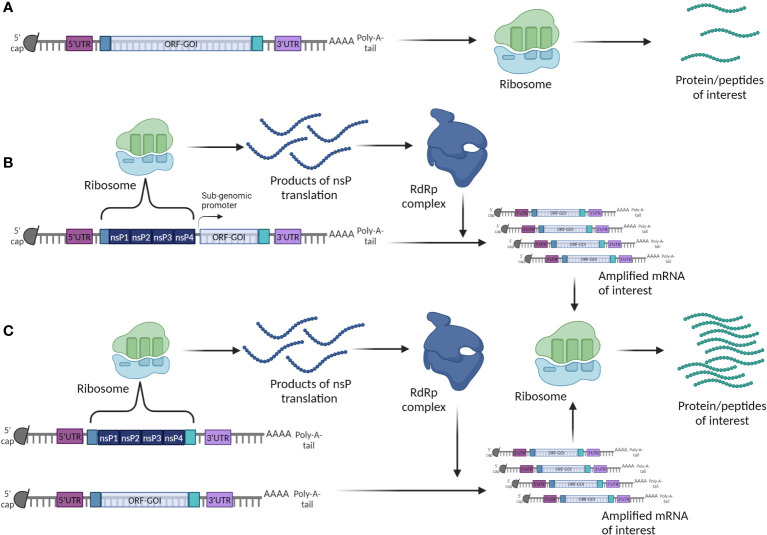
Two strategies to build a mRNA cancer vaccine. **(A)** Conventional or non-replicating mRNA; **(B)** Self-amplifying (replicon) *cis* mRNA; **(C)** Self-amplifying (replicon) *trans* mRNA.

Based on their mechanism of action, mRNA vaccines can be classified as either non-replicating (nrRNA) or self-amplifying (SAM). The latter, on top of the components described above, encode also for the viral replicating machinery (155). The self-amplifying genes of different viral origin encode for non-structural viral replicase proteins (nsPs) that carry information for assembly of the RNA dependent RNA Polymerase complex (RdRp) through which the whole mRNA molecule is amplified. SAM can work on *cis* formation, where both ORFs are being localized on the same mRNA construct, and in *trans* formation, where the ORFs are localized on two different mRNA constructs (156). Therefore, SAM can carry small amounts of mRNA but through amplification can result in high antigen yields (**Figure 8**). Both classes can be used for cancer research, however SAM are more commonly used for infectious diseases (157).

#### 4.3.1 Early clinical trials for mRNA cancer vaccines

mRNA vaccines have shown high efficacy in multiple non-clinical and clinical trials. As a general conclusion, these vaccines are well tolerated and safe to use (158). Currently, most of the anti-cancer mRNA vaccines are at the initial phases of clinical testing (phase I and II clinical trials), and are directed against either TSAs or TAAs.

Recently, a mRNA cancer vaccine (mRNA1457/V940) targeting several TSAs has been developed by Moderna and Merck, and has achieved impressive results in a phase IIb clinical trial (NCT03897881) on cancer patients suffering from resected III/IV stage melanoma. These patients had a high chance of recurrence and had been previously treated with chemotherapy. This vaccine, in combination with pembrolizumab (a PD-1 inhibitor), was able to significantly decrease disease recurrence as compared to pembrolizumab alone, thus highlighting its clinical potential. Interestingly, this vaccine targets up to 34 different neoantigens that can be tailored to individual needs based on tumor molecular landscape (159).

Another mRNA cancer vaccine encoding up to 20 neoantigens has been recently developed by BioNTech, and is called “autogene cevumeran”. This vaccine was used in combination with atelozolizumab (another anti-PDL1), and a chemotherapy regimen comprising four drugs (folinic acid, fluorouracil, irinotecan and oxaliplatin, also called mFOLFIRINOX). The combination was assessed in a phase I clinical trial on patients with resected pancreatic ductal adenocarcinoma (PDAC). Patients that responded positively to the therapy had highly increased levels of long-lived neoantigen specialized T cells targeting multiple neoantigens. In addition, the responders had longer recurrence-free survival suggesting that this vaccine induced efficient anti-tumor immune response (160).

Autogene cevumeran mRNA cancer vaccine in combination with atezolizumab is also been currently tested for the treatment of locally advanced, including metastasized, solid tumors (NCT03289962). In this trial, the effect of the vaccine is being tested in multiple solid tumor types such as melanoma, non-small cell lung cancer (NSCLC), bladder cancer, colorectal cancer, triple negative breast cancer, renal cancer, and head and neck cancer, among others (161). The results of this and other anti-cancer vaccines may completely change the current options for cancer treatments.

In both of the vaccines cited above, neoantigens are selected through sequencing of patient tumor tissue, thus paving the way for personalized treatment. Refinement of this process could further lead to the discovery of new TSAs resulting from specific mutations in specific cancers, thus broadening the use of these vaccines while decreasing their price. Further studies are needed to investigate whether these vaccines can be used as adjuvant and/or neoadjuvant treatments in combination with surgical and chemotherapeutic approaches,

Other companies have chosen the option of targeting TAAs. For instance, Cafri et al. tested a mRNA cancer vaccine encoding up to 20 neoantigens in patients suffering from metastatic gastrointestinal cancer. The patient´s cohort was small, and patients had been treated previously with different therapies including adoptive therapy and immune checkpoint inhibition (ICI). Although immunization was able to induce neoantigen specific immune response and an increase in the number of neoantigen-specific tumor infiltrating lymphocytes, it was not able to improve patient survival (162).

BioNTech developed a cancer mRNA vaccine, called FixVac (BNT 111), targeting simultaneously four melanoma TAAs, including New York esophageal squamous cell carcinoma 1 (NY-ESO-1), melanoma-associated antigen A3 (MAGE-A3), tyrosinase, and transmembrane phosphatase with tensin homology (TPTE) (163). The vaccine was administered intravenously with or without ICI and tested in a phase I clinical trial (NCT02410733) with patients suffering from advanced III/IV stage melanoma that had experienced ICI therapy and had refractory disease. The vaccine was safe to use and induced a significant increase in CD4+ and CD8+ T cells. Currently, this vaccine is being tested in combination or not with cemiplimab (ICI) a in phase II clinical trial (164).

Moderna developed a mRNA cancer vaccine encoding for four highly occurring KRAS mutations including G12C, G12D, G13D, G12V, encapsulated together in LNPs (mRNA-5671/V941) (165). Following promising results in non-clinical studies where this vaccine induced strong cellular immune response against these antigens, this vaccine is currently being tested in a phase I clinical trial (NCT03948763) in NSLC, pancreatic, and colorectal cancer, in combination with pembrolizumab (166).

BioNTech is developing several additional mRNA cancer vaccines targeting different TAAs in various tumor types. For instance, BNT112 mRNA cancer vaccine encodes five TAAs related to metastatic castration-resistant prostate cancer and was tested with or without cemiplimab in a randomized phase I/II clinical trial (NCT04382898). However, this clinical trial was terminated due to sponsor decision (167). Another vaccine from the same company is BNT113, which encodes for anti-human papillomavirus (HPV)-16-derived oncoprotein, and is being tested in HPV16-positive, PD-L1-positive head and neck squamous cell carcinoma in combination with pembrolizumab in a phase II clinical trial (NCT04534205) (168). The same vaccine is being tested in a two-arm dose escalation clinical trial phase I/II in patients with advanced disease and in cancer survivors with no current disease (169). Another mRNA vaccine, BNT115 encodes for several ovarian cancer TAAS and was being tested in ovarian cancer patients, however the recruitment was terminated since the required number of subjects could not be reached (NCT04163094) (170). Furthermore, BNT116 (also known as FixVac) is being tested in a phase I clinical trial (NCT05142189) in patients with metastatic or advanced NSCL cancer in combination with cemiplimab or docetaxel (171).

Besides encoding for tumor antigens, mRNA cancer vaccines are also including the sequence for different immunostimulatory factors such as co-stimulatory molecules or pro-inflammatory cytokines. Most of these vaccines are being administered intratumorally (i.t.).

For instance, Moderna developed a mRNA vaccine (mRNA 2416) that encodes for Oxford 40 ligand (OX-40L) (172). This vaccine passed tolerability and safety tests and demonstrated a potent induction of pro-inflammatory factors in a phase I clinical trial. However, the company decided to terminate this project and proceed with another cancer vaccine named 2752. In phase I clinical trial (NCT03739931), the mRNA cancer vaccine 2752 encoding OX40L/IL23/IL36g is being tested in combination with durvalumab, a PD-L1 inhibitor, in relapsed/refractory solid tumor malignancies or lymphoma (173). BioNTech also developed a mRNA cancer vaccine (SAR441000) encoding for interleukin 12 (IL-12sc), interleukin 15 (IL-15sushi), interferon α (IFNα) and GM-CSF. The vaccine was tested, either alone or in combination with cemiplimab, in patients with advanced solid tumors. However, the most recent updates suggest that the trial was terminated due to non-safety reasons (174).

Another complex candidate is ECI-006, which carries a combination of three mRNAs for dendritic cell (DC) activating molecules such as CD40L, CD70 and caTLR4 (TriMix), and several mRNAs encoding for melanoma TAAs such as tyrosinase, gp100, MAGE-A3, MAGE-C2, and PRAME. This vaccine was being administered intranodally and tested for safety in a phase I clinical trial in patients with resected melanoma (NCT03394937). However, according to newest updates, this trial was terminated due to expiry of medication (175).

Very recently, Gomez et al. described a new cancer vaccine based on RNA-lipid particle aggregates (RNA-LRP) with expanded loading capacity that allows loading of mRNA encoding for glioma-associated antigens (pp65) and a whole tumor RNA, thus significantly increasing its immunogenicity (176). This vaccine was delivered i.v. and tested in immunotherapy-refractory glioblastoma patients in a phase I clinical trial where it was able to induce potent cellular anti-tumor immune response featuring rapid cytokine/chemokine release, as well as immune activation/trafficking, with one patient being in pseudo-progression. The results of this trial indicate that use of multilamellar RNA-LRP, instead of classic LNPs, and i.v. administration can be crucial for targeting characteristically ‘cold’ tumors and for modifying the TME.

In conclusion, currently there is a huge amount of investment and development effort in this field, highlighting the high promise of this therapy.

#### 4.3.2 The advantages of mRNA vaccines over DNA

Several features of the mRNA vaccine approach proved essential and advantageous as compared to other vaccine types. As compared to other vaccination platforms, mRNA vaccines can be designed and produced in a labor-saving and relatively simple way. They are relatively easy to prepare, inexpensive and can be produced in large quantities due to the high yields of IVT technologies (177). Although rapid, the production of these vaccines can be done under good manufacturing practice conditions, as was clearly shown during the SARS-COVID-19 pandemic (145). Since these vaccines are delivered to and processed in the cell cytoplasm, they overcome potential complications related to delivery into the nucleus, namely the potential insertion of foreign DNA into the genome. This is a major difficulty encountered with DNA vaccines. Also, since no viral vectors are used, any potential risk of accidental insertional mutagenesis is reduced (178).

Furthermore, mRNA vaccines mediate a rapid and transient protein expression due to rapid degradation by intracellular mechanisms. In the case of plasmid DNA or viral vectors, the presence of the protein products is much longer, thus allowing the possibility of potential auto-immune complications (179). In addition, the immunogenicity and expression of the mRNA vaccines can be optimized by well-known mechanisms, which further improve their safety (180). Very importantly, as compared to plasmid DNA, mRNA vaccines can target both cells that are actively dividing and the ones that are not, improving the number of cells that can be recruited for antigen generation and presentation (181). In addition, mRNA vaccines are good platforms for personalized therapies, due to their high flexibility, and rapid translatability, once the tumor sequencing results are known. This can improve their efficiency and can be determinant in overcoming difficulties imposed by inter-individual heterogeneity of cancer patients (45). Importantly, they avoid potential anti-vector immunity since they do not need a carrier vector (182).

#### 4.3.3 Side effects

Unexpected toxicity or off-target effects are the main negative consequences of any clinical treatment. Regarding the nature of angiogenesis, there are a number of potential side effects that need to be addressed by these vaccines. These include problems with wound healing, reproductive consequences and a potential inhibition of all physiological processes where angiogenesis is relevant. Fortunately, these processes are relatively limited in adult and elderly cancer patients, but such therapies should be altogether avoided in pregnant women and in developing children, where angiogenesis is rampant. Interestingly, many of the studies presented above have shown lack of these side effects, demonstrating that vaccine blockade of angiogenesis does not necessarily implies problems in wound repair and/or reproduction. This may be due to the fact that tumor angiogenesis often produces rather disorganized, imperfect, and unstable blood vessels (19, 20), which may be the main targets for the vaccines whereas the physiologically well-organized angiogenic process involved in wound healing may not be affected by the vaccines. Nevertheless, caution should be maintained with any new antiangiogenic vaccine product.

New and unexpected information on RNA vaccine behavior is coming up. For instance, a recent study published by Mulroney et al. reports that the presence of modified ribonucleotides in the vaccine RNA can lead to formation of unintended proteins in target cells that could, at least in theory, have different roles from the intended protein. Although it is not suggested that these changes are potentially hazardous, further investigation is needed to learn about all the consequences of this process *in vivo* (183). Furthermore, a paper by Domazet-Lošo suggested the theoretical possibility of retroposition, i.e. the transformation of any RNA molecule into DNA fragments that are inserted into new genomic positions, something that should be taken into account when assessing long term effects of these vaccines (184).

## 5 Conclusion

Over the years, modulating the angiogenesis process has gained importance for the successful treatment of cancer, often in combination with other therapies. Starting with the seminal studies of Judah Folkman (28), the scientific community recognized the potential of this process as a cancer therapy target. Drugs based on monoclonal antibodies and in specific kinase inhibitors have provided useful tools that are now commonly used in the fight against cancer and in other angiogenesis-related fields, such as ophthalmology. On the other hand, the complexity of the angiogenesis process and the limitations associated to the current therapies have prompted the development of new approaches, such as cancer vaccines. These vaccines can target multiple angiogenesis factors/receptors or multiple epitopes of the same factor/receptor and induce a strong immune response that can overcome immune tolerance and other compensation mechanisms. The nucleic acid cancer vaccines, such as those based on DNA and mRNA, are very promising options that could achieve personal and efficient treatments while also being simple, affordable, and rapidly modifiable.

The DNA cancer vaccines were successful in multiple non-clinical studies in targeting tumor-related angiogenesis and vasculature markers. However, in the clinical settings, these vehicles were not able to achieve high success by themselves, but have achieved good results in combination with targeted therapy (monoclonal antibodies). A possibility is that these vaccines were not immunogenic enough to overcome immune tolerance mechanisms and induce immune response against these self-antigens.

However, it must be noted that these vaccines were tested in patients with highly advanced metastatic disease, characterized by impaired immune system functions and the presence of an immune-suppressive environment in the tumors, thus possibly decreasing/inhibiting the immune function. Today, a promising alternative is found in mRNA cancer vaccines. The mRNA approach has been explored for the past 30 years in the design of cancer vaccines, with numerous studies demonstrating feasibility of this approach for cancer treatment (152–171). mRNA vaccines are safe, simple, and highly efficient vehicles that can be optimized and can induce a potent anti-tumor immune response (139). These vaccines have shown in several clinical trials their ability to induce efficient anti-tumor immune responses against different cancer markers, either alone or in combination with other therapeutics, thus confirming their high immunogenic potential. Furthermore, the mRNA platform can be modified rapidly therefore providing great opportunities to adapt to potential changes in the targets/antigens and to keep pace with rapid changing tumors which may become resistant to treatments. This can be potentially crucial in the fight against cancer since it has been shown that a high number of anti-cancer therapeutics, including vaccines, fail due to loss of tumor antigen or downregulation of their expression (185).

Given the current rapidly evolving knowledge on RNA cancer vaccines, we predict this technology will fully revolutionize the way in which cancer patients are treated today.

## Author contributions

ST: Conceptualization, Data curation, Formal analysis, Writing – original draft, Writing – review & editing. AM: Conceptualization, Data curation, Formal analysis, Funding acquisition, Writing – original draft, Writing – review & editing.
