# Supplementary material for: Nucleic acid cancer vaccines targeting tumor related angiogenesis. Could mRNA vaccines constitute a game changer?
Source: Front Immunol. 2024 Jul 16;15:1433185. doi: 10.3389/fimmu.2024.1433185 (PMC11286457; doi:10.3389/fimmu.2024.1433185)
Supplement: Supplementary file 1 [file DataSheet_1.pdf]

Supplementary Material

1 Supplementary Tables

Antigen	Approach	Administration route	Type of tumor model	Tumor model setting	Result	Side effects	References
VEGF	cDNA encoding for homologous *Xenopus laevis* VEGF	i.m.	Fibrosarcoma<br>Breast<br>Hepatoma	Prophylactic<br>Therapeutic	Tumor growth delay; reduction of tumor size	N/A	[86]
MMP-7 and IL-18	DNA cancer vaccines consisting of two plasmids, one encoding for canine MMP-7 and the other one for mouse IL-18. Used in combination or alone	i.m.	Breast	Prophylactic	Tumor growth impairment; reduction of size and weight of tumor; decrease in microvessel density	No side effects were reported	[87]
MMP-2	cDNA encoding for chicken homologous MMP-2	i.m.	Fibrosarcoma<br>Hepatoma<br>Lung	Prophylactic<br>Therapeutic	Reduction of tumor growth ; size reduction of metastases	No side effects reported	[88]
Angiomotin	cDNA encoding for human angiomotin; and in combination with DNA plasmid encoding for human Her-2	i.m.	Breast	Prophylactic	Impairment of tumor growth in transplantable tumor model;<br>Impairment of tumor growth in transgenic model only in combination with Her-2 DNA vaccine	No side effects reported	[89]
Angiomotin	cDNA encoding for human angiomotin	i.m.	Breast	Prophylactic<br>Therapeutic	Impairment of tumor growth in transplantable tumor model;<br>Impairment of tumor growth in transgenic model	No side effects reported	[90]
Tie-2	cDNA encoding for modified MHC I epitope of Tie-2 homologous for human and mouse	i.m.	Not tested	Not tested	Induced antibody specific immune response	No side effects reported	91
DLL-4	cDNA encoding for human DLL-4	i.m. followed by electroporation	Breast	Prophylactic	Tumor growth impairment and delay	No side effects reported	[92]

TEM-1	cDNA encoding for fusion product of mouse TEM-1 and *TTX*-C terminal	i.m.	Colon<br>Lung	Prophylactic<br>Therapeutic	Tumor growth reduction in both models	No side effects reported	[93]
TEM-8	cDNA encoding for human TEM-8 incorporated into attenuated *S. typhimurium*	Orally	Melanoma<br>Lung	Therapeutic	Tumor growth reduction and reduction of metastasis dissemination	No side effects reported	[94]
Endoglin	cDNA encoding for murine endoglin carried by attenuated *S. typhimurium*	Orally	Breast	Prophylactic	Tumor growth impairment and delay; reduction of number and size of metastases	N/A	[95]
Endoglin	cDNA encoding for murine endoglin carried by attenuated *S. typhimurium* and applied in combination or not with cDNA encoding for IL-12, or with cyclophosphamide	Orally	Melanoma<br>Lung	Prophylactic<br>Therapeutic	Tumor growth impairment and delay; size reduction	No side effects reported	[96]
Endoglin	DNA vaccines which consisted of different murine endoglin sequence regions fused to Lysteriolysin O sequence and carried by *L. monocytogenes*	i.p.	Breast	Therapeutic	Tumor growth impairment and delay; size reduction of number and size of metastases	N/A	[97]
FGFR-1	cDNA encoding for FGFR-1 of *X. laevis*	i.m.	Fibrosarcoma<br>Hepatoma<br>Breast	Prophylactic<br>Therapeutic	Tumor growth delay and reduction in both models	Delayed wound healing	[98]
PDGFRβ	cDNA encoding murine PDGFRβ, carried by attenuated *S. typhimurium*	Orally	Colon<br>Lung<br>Breast	Prophylactic<br>Therapeutic	Tumor growth impairment and delay; size reduction of number and size of metastases	No side effects reported	[99]
VEGFR-2	cDNA encoding for murine VEGFR-2 incorporated into vector and carried by *S. typhimurium*	Orally	Colon<br>Lung	Prophylactic<br>Therapeutic	Tumor growth reduction and delay; size reduction of metastases	Delayed wound healing but no effect on reproduction	[100]
VEGFR-2	cDNA encoding for fusion product of murine VEGFR-2 and C3d	i.m.	Bladder	Prophylactic	Tumor growth reduction	N/A	[101]
VEGFR-2	cDNA encoding for fusion product of murine VEGFR-2 and MBD-2 carried by cationic liposome	i.m.	Bladder	Prophylactic<br>Therapeutic	Tumor growth impairment and delay; size reduction of number and size of metastases	N/A	[102]

VEGFR-2	cDNA encoding for murine VEGFR-2 carried by *S. typhimurium* in combination or not with cDNA encoding for IP-10	Orally and i.t.	Melanoma	Prophylactic	Impairment of tumor growth in transplantable tumor model	No side effects reported	[103]
VEGFR-2	Attenuated *S. typhymurium* coated with self-assembled nanoparticles made of a cationic polymer, beta cyclodextrin polyethylenimine 600 (PEI600-β-CyD) and cDNA encoding for murine VEGFR-2	Orally	Melanoma	Prophylactic	Impairment of tumor growth in transplantable tumor model	N/A	[104]
VEGFR-2	Plasmid encoding for fusion gene product of human *VEGFR-2* and *IL-12*	i.m. followed by electroporation.	Melanoma	Prophylactic	Tumor growth impairment; reduction of size and weight of tumor	N/A	[105]
VEGFR-2 and E6E7	Fusion vaccine targeting E6E7 HPV markers and human VEGFR-2	i.m. followed by electroporation	Lung	Therapeutic	Tumor growth impairment; reduction of size and weight of tumor	No side effects were reported	[106]
VEGFR-2	Plasmid encoding for human VEGFR-2	i.d. followed with electroporation	Lung<br>Breast	Prophylactic<br>Therapeutic<br>Therapeutic with primary tumor surgical removal	In prophylactic setting decrease in tumor size and impairment of tumor growth correlated with longer median survival of animals. In therapeutic setting, no effect on tumor growth was observed, and immunization did not improve survival of animals.	Induced liver micrometastasis in B16-F10 model after the immunization; No other side effects were observed.	[107]
VEGFR-2	Plasmid encoding for human VEGFR-2	i.m. followed with electroporation	Not tested	Not tested	In both increase in humoral and cellular immune response were observed after the immunization. *In vitro* cytotoxicity was observed in mouse after the incubation of PBMCs with tumor cells.	No side effects were reported	[108]
VEGFR-3	Plasmid encoding for murine VEGFR-3 carried by attenuated *S. typhimurium*	Orally	Lung	Prophylactic<br>Therapeutic	Tumor growth impairment; reduction of tumor volume; longer median survival; decrease in tumor lymph vessel density.	N/A	[109]

Survivin	cDNA encoding for mouse survivin and a chemokine CCL21 carried by attenuated *S. typhimurium*	Orally	Lung	Prophylactic<br>Therapeutic	Tumor growth impairment and delay; prevention of metastastic formation in prophylactic model; size reduction of number and size of metastases in therapeutic model	No side effects reported	[110]
Survivin	DNA based replicon vaccine derived from SFV containing sequence for human survivin and/ or hCG and additionally for, B7, IgGFc and GM-CSF.	i.m. followed by electroporation	Melanoma<br>Lung	Prophylactic<br>Therapeutic	Tumor growth impairment and delay; size reduction of number and size of metastases.	No side effects reported	[111]
Survivin and VEGFR-2	DNA based replicon vaccine derived from SFV containing sequence for human survivin and/ or hCG and additionally for B7,IgGFc,<br>GM-CSF combined with the same vaccine coding for human VEGFR-2 and IL-12.	i.m. followed by electroporation	Melanoma<br>Lung	Prophylactic<br>Therapeutic	Tumor growth impairment and delay; reduction in number and size of metastases.	No side effects reported	[75]
Survivin and MUC-1	DNA based vaccine constructed of bicistronic vector coding for human survivin and MUC-1, but also for human IL-2 and CpG found in the vector backbone. Tested additionally along with oxiplatin.	i.m. for DNA vaccine and i.p. for chemotherapy	Colorectal	Therapeutic	Tumor growth impairment and delay; reduction in number and size of metastases. Prolonged median survival time.	No side effects reported	[112]
Survivin, MUC-1 and sPD-1	DNA based vaccine constructed of bicistronic vector coding for human survivin and human MUC-1, but also for sPD1. As adjuvants, human IL-2 and CpG found in the vector backbone were used. Tested additionally along with oxiplatin	i.m. for DNA vaccine and i.p. for chemotherapy	Colorectal<br>Lung	Therapeutic	Tumor growth impairment and delay	N/A	[113]
Survivin and FAPα	Fusion DNA vaccine targeting human survivin and human FAPα, with incorporated CpG motif as adjuvant.	i.m. for DNA vaccine followed with electroporation and i.p. for chemotherapy	Breast	Therapeutic	Tumor growth impairment and delay; reduction in number and size of metastases.	N/A	[114]

					Prolonged median survival time.		
Survivin	A minigene DNA vaccine targeting human survivin.	i.m	Neuroblastoma	Prophylactic<br>Therapeutic	Tumor growth impairment and delay; reduction in number and size of metastases. Prolonged median survival time.	No side effects were reported.	115
Survivin	Plasmid coding for phosphorilation-defective mouse survivin T34-A was encapsulated into liposome consisting of cholesterol and DOTAP.	i.v.	Breast	Therapeutic	Tumor growth impairment; reduction in number of metastases; decrease in microvessel density.	No side effects were reported.	116
Survivin	Plasmid coding for phosphorilation-defective mouse survivin T34-A was encapsulated into liposome consisting of cholesterol and DOTAP. Used in combination or not with cisplatin.	i.v.	Lung	Therapeutic	Tumor growth impairment; reduction in number of metastases; decrease in microvessel density	N/A	117
Survivin	Plasmid coding for phosphorylation-defective mouse survivin T34-A was encapsulated into liposome consisting of cholesterol and DOTAP. Used in combination or not with radiotherapy.	i.v.	Lung	Therapeutic	Tumor growth impairment; reduction in number of metastases; decrease in microvessel density.	N/A	118
Survivin	Plasmid coding for phosphorylation-defective mouse survivin T34-A was encapsulated into liposome consisting of DOTAP.	i.p.	Cervical	Therapeutic	Tumor growth impairment; reduction in number of metastases; decrease in microvessel density.	N/A	119
Survivin	Plasmid coding for phosphorylation-defective mouse survivin T34-A was encapsulated into liposome consisting of cholesterol DOTAP.	i.v.	Prostate	Therapeutic	Tumor growth impairment; reduction of size and weight of tumor; decrease in microvessel density.	No side effects were reported	120
Survivin and hCG	Combination of two DNA cancer vaccines. Two plasmids, one coding for a fusion gene product of human survivin and hCG and	i.m. followed by electroporation	Breast	Therapeutic	Tumor growth impairment; reduction of size and weight of tumor; decrease in microvessel density.	No side effects were reported	121

	the other one coding for an IL-12 adjuvant.				Combination of vaccines had superior effect as compared to any of vaccines alone.		
Survivin	Two naked DNA vaccine against secreted and non-secreted human survivin, with additional injection of naked DNA vaccine targeting mouse GM-CSF as adjuvant.	i.m.	Not tested	Not tested	Induced both humoral and cellular immune response. Main cell subpopulation involved in cellular response were CD8 IFNy-positive T cells while humoral was mainly Th1 CD4+ directed.	N/A	122
Survivin	Naked DNA vaccine encoding an *in silico* predicted CD8+ T cell epitope of human survivin	i.d. electroporation	Melanoma	Prophylactic<br>Therapeutic	Induced CD8+ IFNy-positive cell immune responses and impeded tumor-related angiogenesis; tumor growth impaired in both settings; in prophylactic setting tumor regression was observed and animals remained tumor-free.	N/A	123

Supplementary Table 1. Summary of non-clinical studies targeting angiogenic factors with DNA vaccine technology. N/A: Not Available, VEGF: Vascular endothelial growth factor, cDNA: complementary DNA, i.m.: intramuscular, i.t.: intratumoral, MMP-7: Matrix metalloproteinase 7, IL-18: Interleukin 18, MMP-2: Matrix metalloproteinase 2, Tie-2: angiopoietin-1 receptor, DLL-4: Delta-like ligand 4, TEM-1: tumor endothelial marker 1, TEM-8: tumor endothelial marker 8, IL-12: Interleukin 12, i.p.: intraperitoneal, FGFR-1: Fibroblast growth factor receptor 1, PDGFRβ: platelet derived growth factor receptor β, VEGFR-2: Vascular endothelial growth factor 2, C3d: complement component 3d, MBD-2: murine beta defensin 2, IP-10: interferon-induced protein-10, PBMCs: Peripheral blood mononuclear cells, VEGFR-3: Vascular endothelial growth factor 3, IgGFc: immunoglobulin G Fc fragment, GM-CSF: Granulocyte-macrophage colony-stimulating factor, sPD-1: soluble PD-1, DOTAP: 1,2-dioleoyl-3-trimethylammonium-propane, T34-A:survivine threonine 34 alanine mutant, hCG: human chorionic gonadotropin, HPV: Human papilomavirus, SFV: Semiliki Forest virus, FAPα: fibroblast activating protein alpha.

Antigen	Approach	Administration route	Type of tumor model	Tumor model setting	Result	Side effects	References
VEGFR -2	cDNA encoding for human VEGFR-2 carried by *S. typhimurium*	Orally	Advanced pancreatic cancer	Therapeutic	Elevation of antigen-specific effector T cells levels; reduction of tumor perfusion	Transient side effects - Decrease in number of lymphocytes	[125]
VEGFR -2	cDNA encoding for human VEGFR-2 carried by *S. typhimurium*	Orally	Advanced pancreatic cancer	Therapeutic	Elevation of antigen-specific effector T cells levels	Transient side effects - Decrease in number of lymphocytes, diarrhea	[126]
VEGFR -2	cDNA encoding for human VEGFR-2 carried by *S. typhimurium* in combination or not with nivolumab.	Orally and nivolumab via i.v.	Progressive glioblastoma	Therapeutic	Elevation of antigen-specific effector T cells levels; Favorable outcome in 5 out of 14 patients with 2 out of 5 with complete response in two years.	Transient side effects; Decrease in number of lymphocytes, diarrhea. Concluded that most of the side effects were not direct consequence of therapy but rather result of cancer patient health state.	[127]
VEGFR -2	cDNA encoding for human VEGFR-2 carried by *S. typhimurium* in combination or not with avalumab.	Orally and avalumab via i.v.	Progressive glioblastoma	Therapeutic	Elevation of antigen-specific effector T cells levels; 3 out of 9 patients partial response was observed and two of these patients at the moment of the report publishing were more than 6 months progression free.	Transient side effects	[128]

Supplementary Table 2. Summary of published studies reporting clinical trials on DNA vaccines directed against angiogenic targets. VEGFR-2: Vascular endothelial growth factor receptor 2, cDNA: complementary DNA, i.v.: intravenous.
